# Identification of Hypolipidemic Constituents from *Microctis folium* Using an Integrated Strategy of Chemical Profiling, Pharmacokinetics, Network Pharmacology and Experimental Validation

**DOI:** 10.3390/ph19091349

**Published:** 2026-08-26

**Authors:** Zhihao Zeng, Yanchang Liu, Xiaoli Bi, Wanchun Chen, Yunhui Ouyang, Jingnian Zhang, Weitao Chen, Guanlin Xiao

**Affiliations:** 1 Department of Traditional Chinese Medicine Analysis, Guangdong Provincial Engineering Technology Research Institute of Traditional Chinese Medicine, Guangzhou 510095, China; zengzhihao129@126.com (Z.Z.); zyfxyjs@gzucm.edu.cn (X.B.); zjn9110@163.com (J.Z.); chenwt009@126.com (W.C.); 2 Guangdong Provincial Key Laboratory of Research and Development in Traditional Chinese Medicine, Guangzhou 510095, China; 3 The Fifth Clinical College of Guangzhou University of Chinese Medicine, Guangzhou 510095, China; lyc198683841142023@163.com (Y.L.); zxzxas202012@163.com (W.C.);

**Keywords:** *Microctis folium*, hyperlipidemia, UPLC fingerprint, pharmacokinetics, network pharmacology

## Abstract

**Background/Objective**: Traditionally derived from the dried leaves of *Microcos paniculata* L., *Microctis folium* (*M. folium*) has been employed to manage various inflammatory and metabolic conditions. Despite its reported hypolipidemic potential, the active constituents contributing to this effect and their molecular mechanisms of action remain to be determined. Therefore, this study aimed to systematically identify the active hypolipidemic constituents of *M. folium* and elucidate their mechanisms of action through an integrated strategy combining chemical profiling, pharmacokinetic screening, network pharmacology, and experimental validation. **Methods**: UPLC fingerprint and UPLC-QQQ-MS/MS were employed to characterize and quantify the chemical constituents of *M. folium*. Pharmacokinetic analysis was conducted to identify systemically absorbed compounds. Network pharmacology was applied to predict potential targets and signaling pathways. The lipid-lowering effects were subsequently validated *in vitro* using an oleic acid/palmitic acid (OA/PA)-induced lipid accumulation model in HepG2 cells and *in vivo* in a Triton WR-1339-induced hyperlipidemia mouse model. **Results**: UPLC fingerprint analysis identified 15 common peaks among 21 batches of *M. folium*, with similarity values ranging from 0.885 to 0.990, indicating good chemical consistency. Eighteen representative compounds were quantified, among which flavone C-glycosides and phenolic acids were predominant. Pharmacokinetic results demonstrated that multiple *M. folium*’s constituents were absorbed into systemic circulation. Network pharmacology analysis identified 73 potential targets related to hyperlipidemia and highlighted the PI3K-Akt signaling pathway as a key regulatory pathway. *In vitro* experiments showed that *M. folium*’s compounds significantly reduced intracellular lipid accumulation and oxidative stress in OA/PA-induced HepG2 cells. Furthermore, *in vivo* studies demonstrated that vitexin, ferulic acid, isoferulic acid, and *N*-trans-feruloyltyramine significantly reduced serum and hepatic lipid levels, including TC, TG, and LDL-c, alleviated hepatic steatosis, and improved liver injury and oxidative stress markers in Triton WR-1339-induced hyperlipidemic mice. **Conclusions**: *M. folium* exerts significant lipid-lowering effects through multi-component and multi-target mechanisms involving regulation of lipid metabolism and oxidative stress, partly mediated by activation of the PI3K-Akt signaling pathway. These findings provide a scientific basis for the development of *M. folium*’s natural agents for the treatment of hyperlipidemia.

## 1. Introduction

Hyperlipidemia is a major metabolic disorder characterized by elevated levels of circulating triglycerides and cholesterol and is a key contributor to metabolic-associated fatty liver disease and cardiovascular morbidity [1]. Despite the widespread use of statins and fibrates, long-term pharmacotherapy is frequently associated with adverse reactions and limited efficacy in complex metabolic conditions [2]. Therefore, increasing attention has been directed toward natural products with multi-target regulatory properties that may provide complementary or alternative therapeutic strategies [3,4].

Plant-derived flavonoids and phenolic acids have demonstrated promising lipid-lowering, antioxidant, and anti-inflammatory activities. Accumulating evidence indicates that these natural products regulate hepatic lipid synthesis, fatty acid oxidation, and inflammatory signaling pathways such as PI3K-Akt and MAPK, which are critically involved in lipid homeostasis [5,6]. In experimental models of diet-induced dyslipidemia, flavonoid supplementation has been shown to attenuate hepatic steatosis and improve serum lipid profiles through coordinated modulation of oxidative stress and metabolic signaling cascades [7,8]. These findings highlight the therapeutic potential of phenolic-rich herbal medicines in metabolic diseases.

*Microctis folium* (*M. folium*), the dried leaves of *Microcos paniculata* L., is widely distributed in southern China and Southeast Asia. Traditionally, *M. folium* has been used for throat inflammation, rheumatic disorders, and hypertension, suggesting its relevance in inflammatory and vascular conditions. Recent phytochemical investigations have revealed that *M. folium* is abundant in flavonoid glycosides and phenolic acids, exhibiting significant antioxidant capacity in radical-scavenging assays [9]. Bioactive constituents such as vitexin, ferulic acid, and related phenolics identified in *M. folium* or phylogenetically related species have been reported to alleviate oxidative injury and metabolic dysfunction [10,11]. However, despite its documented antioxidant and anti-inflammatory activities, the lipid-lowering potential of *M. folium* remains insufficiently characterized, and the specific active constituents responsible for its metabolic effects have not been systematically defined.

Hyperlipidemia is driven by intertwined processes including dysregulated lipid synthesis, excessive reactive oxygen species production, and chronic low-grade inflammation. Considering the multi-target regulatory capacity of phenolic compounds, *M. folium* is likely to exert hypolipidemic effects through the integrated modulation of metabolic and inflammatory pathways. Nevertheless, traditional phytochemical approaches often rely solely on compound isolation or *in vitro* screening, without integrating systemic exposure data or mechanistic target prediction [12]. Such strategies may overlook compounds with favorable pharmacokinetic profiles that are crucial for *in vivo* efficacy.

Recent advances in analytical chemistry, pharmacokinetics, and network pharmacology have provided powerful tools to decipher the material basis and molecular mechanisms of herbal medicines. The integration of ultra-performance liquid chromatography-tandem mass spectrometry (UPLC-MS/MS), *in vivo* pharmacokinetic evaluation, and *in silico* target prediction enables the identification of bioavailable compounds with mechanistic relevance [13,14]. When combined with experimental validation, this systematic approach enhances the reliability of active component screening and mechanistic interpretation.

Therefore, the present study aimed to elucidate the hypolipidemic material basis and molecular mechanisms of *M. folium* using an integrated strategy. UPLC fingerprinting and quantitative analysis were performed to characterize its chemical profile. Pharmacokinetic evaluation was subsequently conducted to identify systemically exposed constituents. Network pharmacology analysis was employed to predict potential targets and pathways, followed by *in vitro* validation using an oleic acid/palmitic acid-induced hepatocyte lipid accumulation model and *in vivo* verification in a hyperlipidemia mouse model. Through this comprehensive approach, we sought to clarify the active constituents and signaling pathways underlying the lipid-regulatory effects of *M. folium*, thereby providing a scientific foundation for their potential development as natural therapeutic agents for hyperlipidemia.

## 2. Results

### 2.1. Establishment and Evaluation of UPLC Fingerprint of M. folium

**Figure 1:**
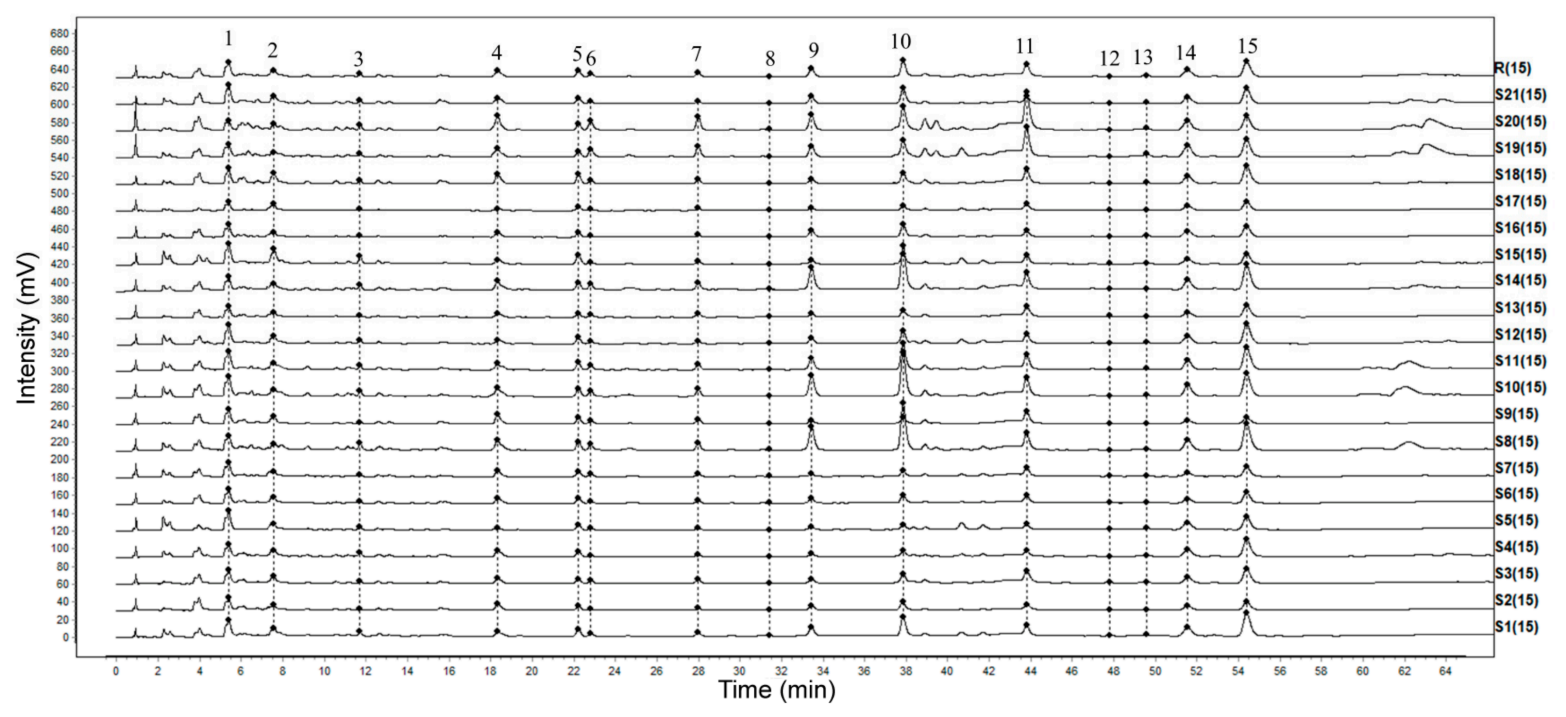
UPLC fingerprint of *M. folium*. 1, 2: Unidentified peaks. 3: Caffeic acid. 4: Cis-p-coumaric acid. 5: Ferulic acid. 6, 7: Unidentified peaks. 8: Schaftoside. 9: Vitexin. 10: Isovitexin. 11: Violanthin. 12: Kaempferol-3-β-*O*-glucuronide. 13: *N*-trans-feruloyltyamine. 14: Unidentified peak. 15: Narcissoside. S1–S21 represent the fingerprints of 21 different batches of *M. folium*, with the corresponding batch numbers provided in Supplementary Table S2. R represents the reference fingerprint generated from the 21 batches of *M. folium*.

UPLC fingerprint method for *M. folium* was successfully established under the optimized chromatographic conditions. Representative chromatograms recorded at 315 nm exhibited good peak resolution, stable baseline separation, and abundant characteristic peaks. Based on the similarity evaluation system for chromatographic fingerprints of traditional Chinese medicine, a total of 15 common peaks were identified among 21 batches of *M. folium* samples. The similarity values of all batches ranged from 0.885 to 0.990, indicating high batch-to-batch consistency and overall stability of the chemical profile (Figure 1 and Supplementary Table S5).

By comparison with reference standards, ten characteristic components were unambiguously identified, including caffeic acid, cis-p-coumaric acid, ferulic acid, schaftoside, vitexin, isovitexin, violanthin, kaempferol-3-β-*O*-glucoside, *N*-trans-feruloyltyramine, and narcissoside. These compounds mainly belonged to phenolic acids, flavone C-glycosides, and flavonol glycosides, suggesting that phenylpropanoids and flavonoids represent the major chemical constituents of *M. folium*.

### 2.2. Quantitative Analysis of Major Constituents by UPLC-QQQ-MS/MS

A sensitive and selective UPLC-QQQ-MS/MS method was developed for the simultaneous quantification of 18 representative compounds in *M. folium*. All analytes showed good chromatographic separation within 15 min and were detected with high sensitivity using MRM in positive or negative ion modes.

Method validation results demonstrated satisfactory linearity for all compounds within their respective concentration ranges, with correlation coefficients greater than 0.99 (Supplementary Table S6). The intra- and inter-day precision values were within acceptable limits, and the accuracy and recovery of all analytes met the requirements of bioanalytical method validation guidelines. These results confirmed that the developed method was reliable for quantitative determination of multiple components in complex herbal matrices.

### 2.3. Contents of Main Compounds in M. folium

The established UPLC-QQQ-MS/MS method was applied to determine the contents of the 18 compounds in 21 batches of *M. folium* (Supplementary Tables S6 and S7). Considerable differences in the absolute contents of individual compounds were observed among batches, while the overall compositional pattern remained consistent, in agreement with the fingerprint analysis.

Among the quantified compounds, schaftoside, vitexin, isovitexin, violanthin, narcissoside, and rutin were present at relatively high levels, indicating that flavone C-glycosides constitute the predominant components of *M. folium*. Phenolic acids such as caffeic acid, ferulic acid, isoferulic acid, and cis-p-coumaric acid were also consistently detected, whereas flavonol glycosides and phenethylamide derivatives showed comparatively lower contents. The quantitative results further supported the reliability of the fingerprint analysis and provided a comprehensive chemical basis for subsequent pharmacological and mechanistic studies.

### 2.4. Integration of Fingerprint and Quantitative Analyses

By combining UPLC fingerprint analysis with UPLC-QQQ-MS/MS quantification, a systematic chemical characterization of *M. folium* was achieved. The fingerprint analysis ensured the overall consistency and integrity of the herbal material, while the quantitative results clarified the distribution and abundance of key bioactive constituents.

Importantly, several high-content compounds identified in this study, particularly flavone C-glycosides and phenolic acids, have been reported to possess lipid-regulating, antioxidant, and anti-inflammatory activities. These findings provide a solid chemical foundation for subsequent screening of active compounds using cell extraction, pharmacokinetic evaluation, and biological verification.

### 2.5. Pharmacokinetic Analysis

**Figure 2:**
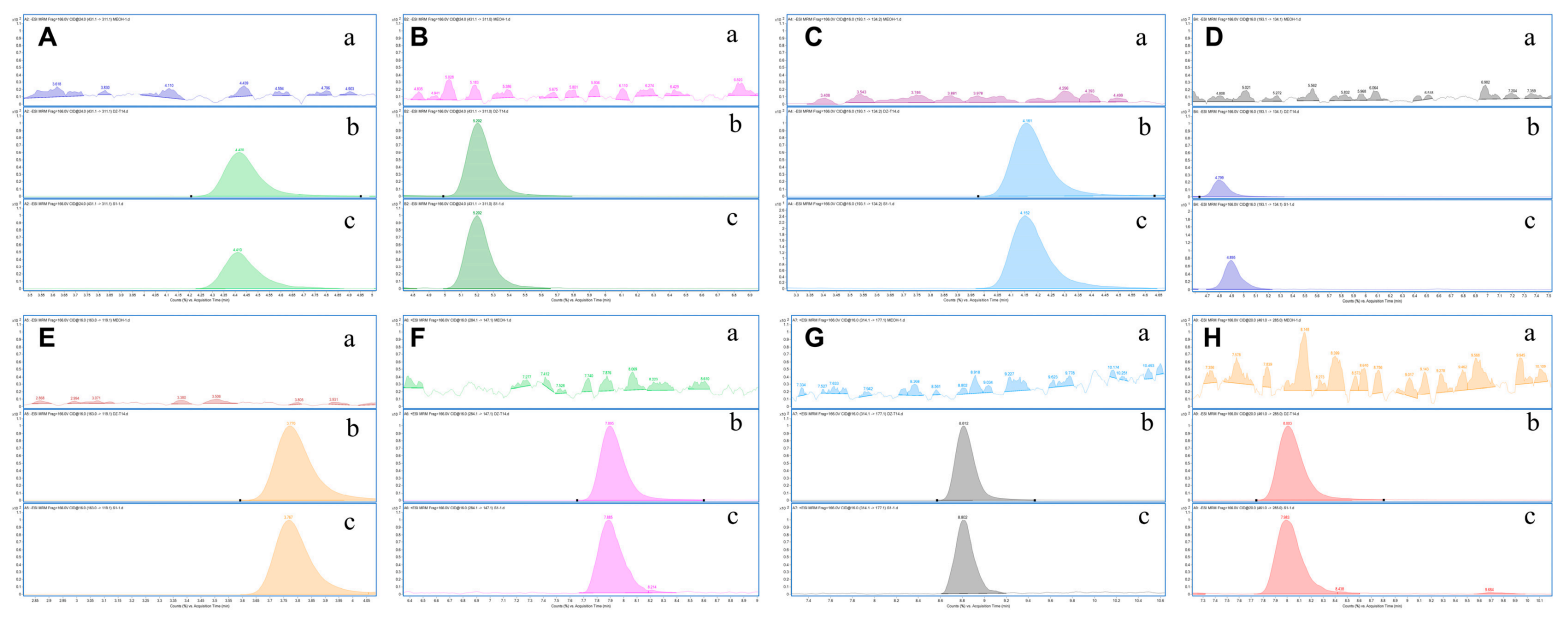
The total ion of eight constituents was absorbed into the systemic circulation. (**A**) Vitexin. (**B**) Isovitexin. (**C**) Ferulic acid. (**D**) Isoferulic acid. (**E**) Cis-p-coumaric acid. (**F**) *N*-trans-feruloyltyramine. (**G**) *N*-p-trans-coumaroyltyramine. (**H**) Kaempferol-3-β-*O*-glucuronide. (**a**) Blank. (**b**) Reference standard. (**c**) Sample.

After oral administration of *M. folium* extract, eight representative compounds were successfully detected in rat plasma using the UPLC-QQQ-MS/MS method, indicating that these compounds were absorbed into the systemic circulation (Figure 2). No endogenous interference was observed at the corresponding retention times in blank plasma samples.

**Figure 3:**
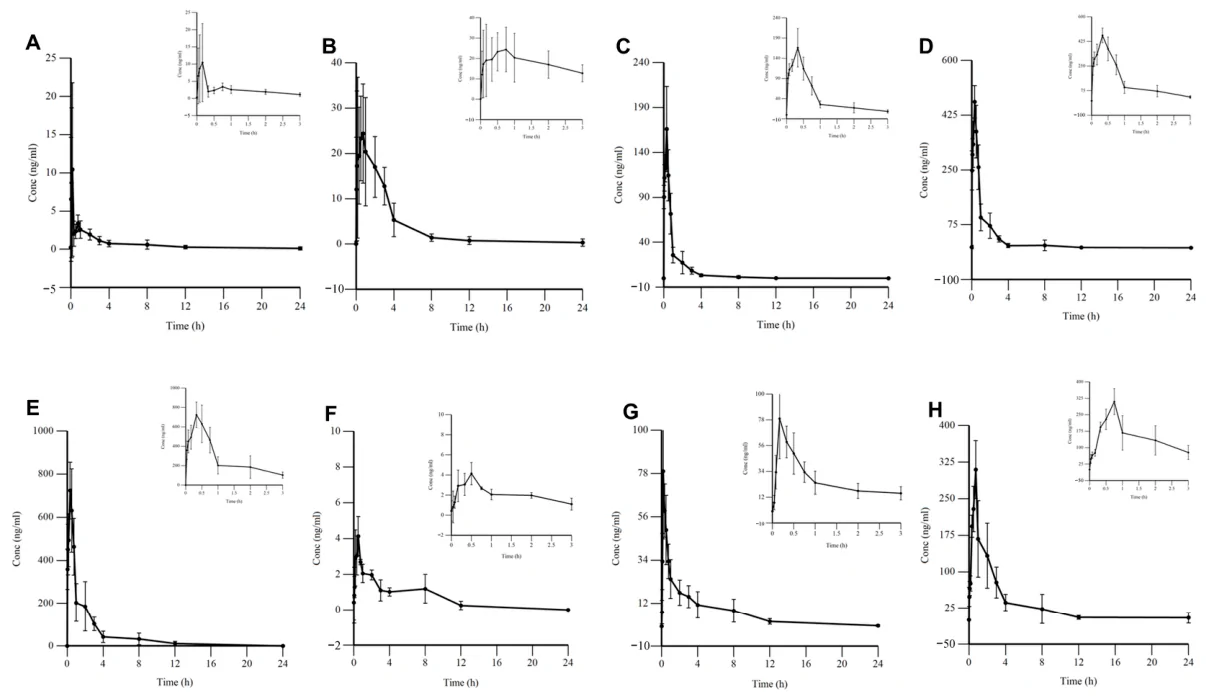
The plasma concentration–time profiles of the eight compounds (*n* = 6). (**A**) Vitexin. (**B**) Isovitexin. (**C**) Ferulic acid. (**D**) Isoferulic acid. (**E**) Cis-p-coumaric acid. (**F**) *N*-trans-feruloyltyramine. (**G**) *N*-p-trans-coumaroyltyramine. (**H**) Kaempferol-3-β-*O*-glucuronide.

The plasma concentration–time profiles of the eight compounds are shown in Figure 3. Most compounds exhibited rapid absorption, reaching peak plasma concentrations within a relatively short time after administration, followed by a gradual decline. However, noticeable differences in absorption rate and systemic exposure were observed among the compounds.

Pharmacokinetic parameters were calculated using a non-compartmental analysis method and are summarized in Table 1 and Supplementary Table S8. Among the eight compounds, flavonoid glycosides such as vitexin and isovitexin showed relatively higher plasma exposure, as indicated by larger AUC values, whereas phenolic acids displayed faster elimination profiles. The T_max_ values of most compounds occurred within the early post-dosing period, suggesting rapid gastrointestinal absorption of *M. folium*-derived constituents.

Overall, the pharmacokinetic results demonstrated that multiple components of *M. folium* extract could be absorbed *in vivo*, although their systemic exposure and elimination behaviors varied considerably. These absorbed compounds were therefore selected as candidate active constituents for subsequent network pharmacology analysis and pharmacodynamic validation.

### 2.6. In Silico Analysis

#### 2.6.1. Potential Targets of Active Compounds and Hyperlipidemia

A total of 212 potential targets were predicted for the eight representative compounds of *M. folium* after target prediction and standardization. Meanwhile, 1381 hyperlipidemia-related targets were collected from the GeneCards, OMIM, and DisGeNET databases after removing duplicate entries.

**Figure 4:**
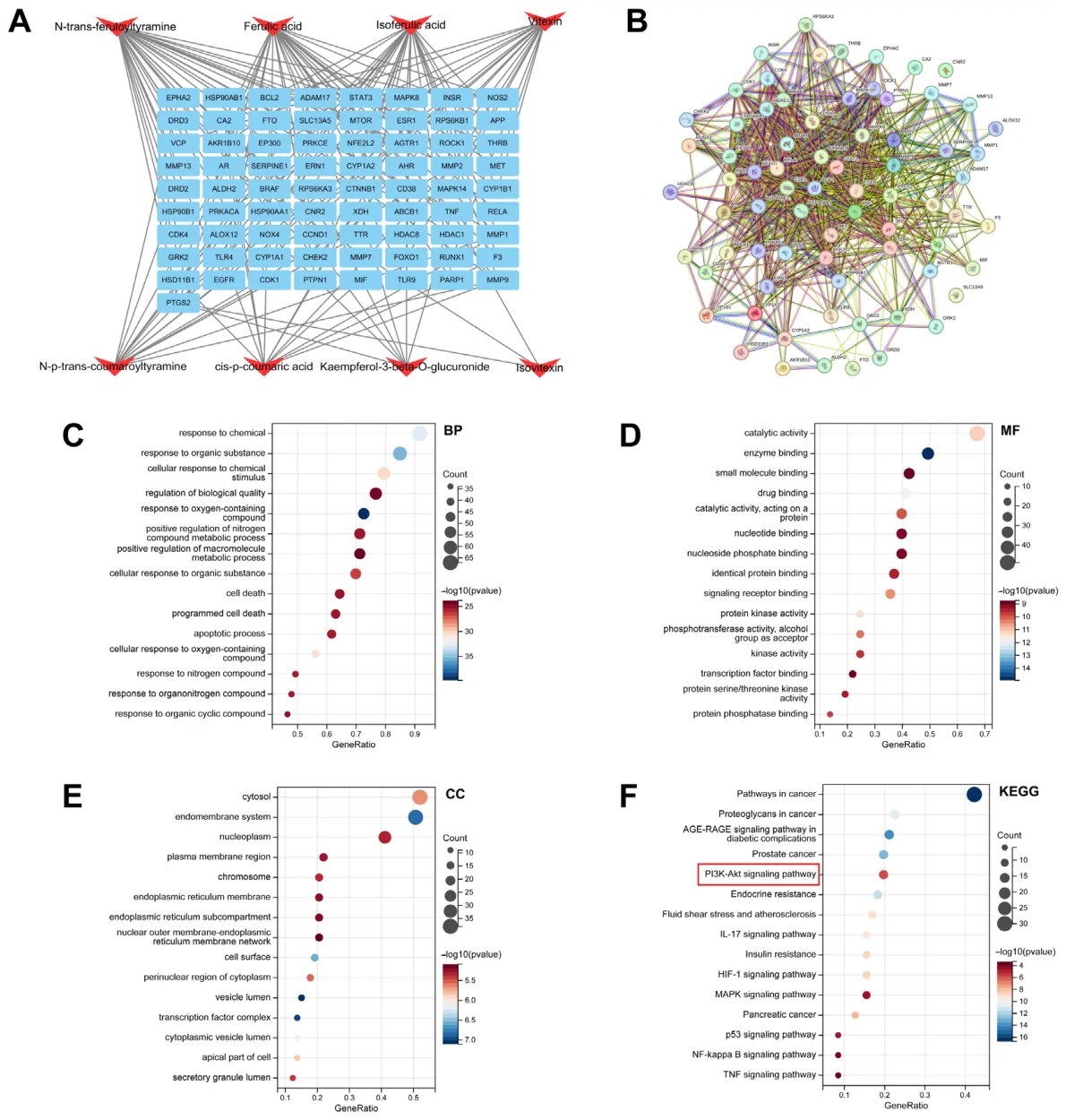
Network pharmacology of *M. folium* in the treatment of hyperlipidemia. (**A**) Diagram of *M. folium* and hyperlipidemia intersection targets. (**B**) PPI network. (**C**) Biological processes. (**D**) Molecular functions. (**E**) Cellular components. (**F**) KEGG pathways. The red box highlights the PI3K-Akt signaling pathway, which was identified as an important pathway in this study.

A total of 73 targets were identified at the intersection of the compound-associated and hyperlipidemia-related targets (Figure 4A), suggesting that *M. folium* may exert its lipid-lowering effects through multiple shared molecular targets.

#### 2.6.2. PPI Network Analysis

The overlapping targets were imported into the STRING database to construct a PPI network (Figure 4B). The network consisted of 73 nodes and 751 edges, indicating extensive interactions among the shared targets.

#### 2.6.3. GO Enrichment Analysis

GO enrichment revealed that the overlapping targets were enriched in biological processes related to lipid metabolism, inflammatory response, oxidative stress, and regulation of cell survival (Figure 4C). The enriched cellular components were mainly associated with membrane structures, cytosol, and protein complexes, while molecular functions were primarily involved in kinase activity, receptor binding, and transcriptional regulation (Figure 4D,E).

#### 2.6.4. KEGG Pathway Enrichment Analysis

KEGG enrichment showed that the overlapping targets were significantly involved in multiple signaling pathways associated with metabolic regulation and inflammation (Figure 4F). Notably, pathways such as PI3K-Akt signaling, MAPK signaling, insulin resistance, and lipid metabolism-related pathways were highly enriched.

These pathways are closely associated with lipid homeostasis and metabolic disorders, suggesting that *M. folium* may exert its anti-hyperlipidemic effects through the coordinated regulation of multiple signaling cascades.

### 2.7. In Vitro Analysis

#### 2.7.1. Effects of *M. folium*-Derived Compounds on HepG2 Cell Viability

**Figure 5:**
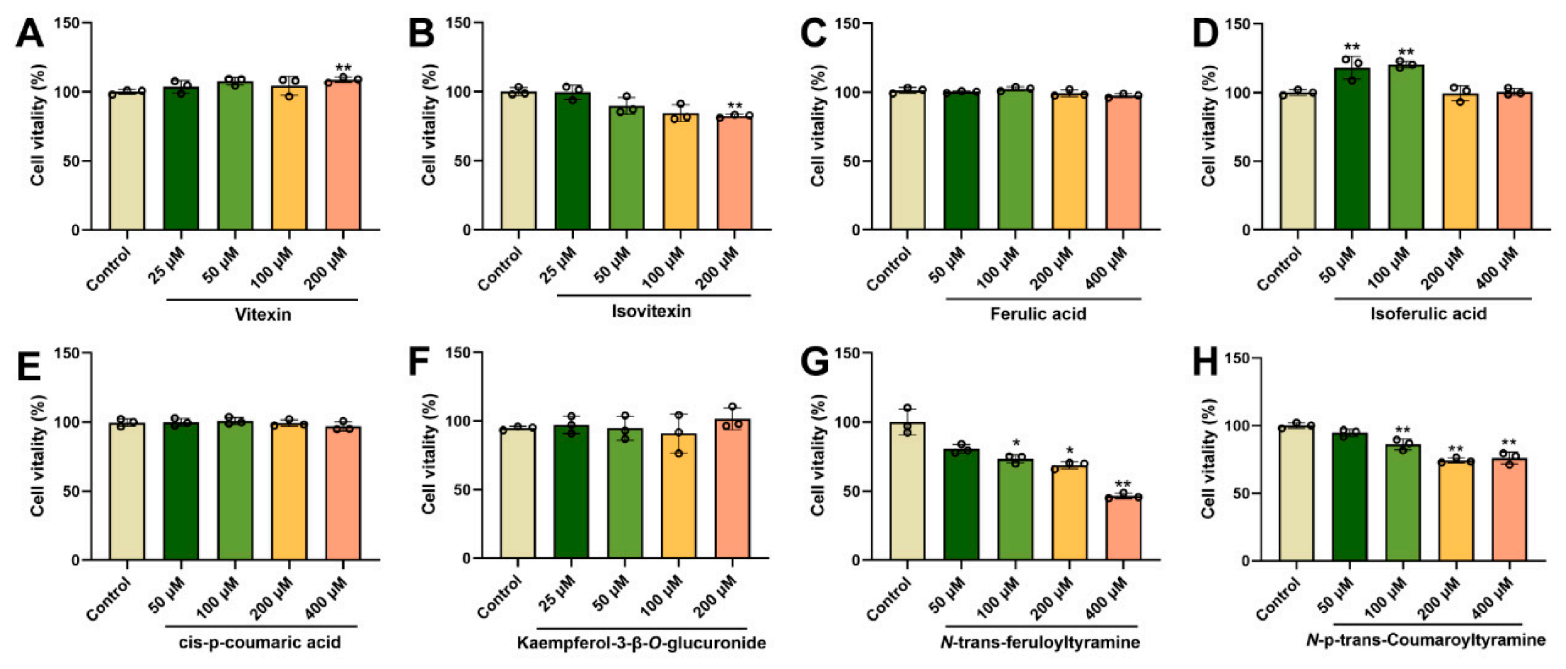
Effects of *M. folium*-derived compounds on HepG2 cell viability (*n* = 3). (**A**) Vitexin. (**B**) Isovitexin. (**C**) ferulic acid. (**D**) Isoferulic acid. (**E**) Cis-p-coumaric acid. (**F**) *N*-trans-feruloyltyramine. (**G**) *N*-p-trans-coumaroyltyramine. (**H**) Kaempferol-3-β-*O*-glucuronide. All data are represented as mean ± SD. * *p* < 0.05 and ** *p* < 0.01 as compared to Control.

The CCK-8 assay determined the cytotoxicity of *M. folium*-derived compounds in HepG2 cells. As shown in Figure 5, treatment with vitexin, isovitexin, ferulic acid, isoferulic acid, cis-p-coumaric acid, kaempferol-3-β-*O*-glucuronide, and *N*-p-trans-coumaroyltyramine at the tested concentrations showed no significant cytotoxic effects on HepG2 cells. However, *N*-trans-feruloyltyramine exhibited a slight reduction in cell viability at higher concentrations, although cell viability remained above 70%. Based on these results, appropriate concentrations were selected for the subsequent experiments, including vitexin (200 μM), isovitexin (200 μM), ferulic acid (400 μM), isoferulic acid (400 μM), cis-p-coumaric acid (400 μM), kaempferol-3-β-*O*-glucuronide (200 μM), *N*-trans-feruloyltyramine (50 μM), and *N*-p-trans-coumaroyltyramine (50 μM).

#### 2.7.2. Effects of *M. folium* on Lipid Accumulation and Oxidative Stress in HepG2 Cells

**Figure 6:**
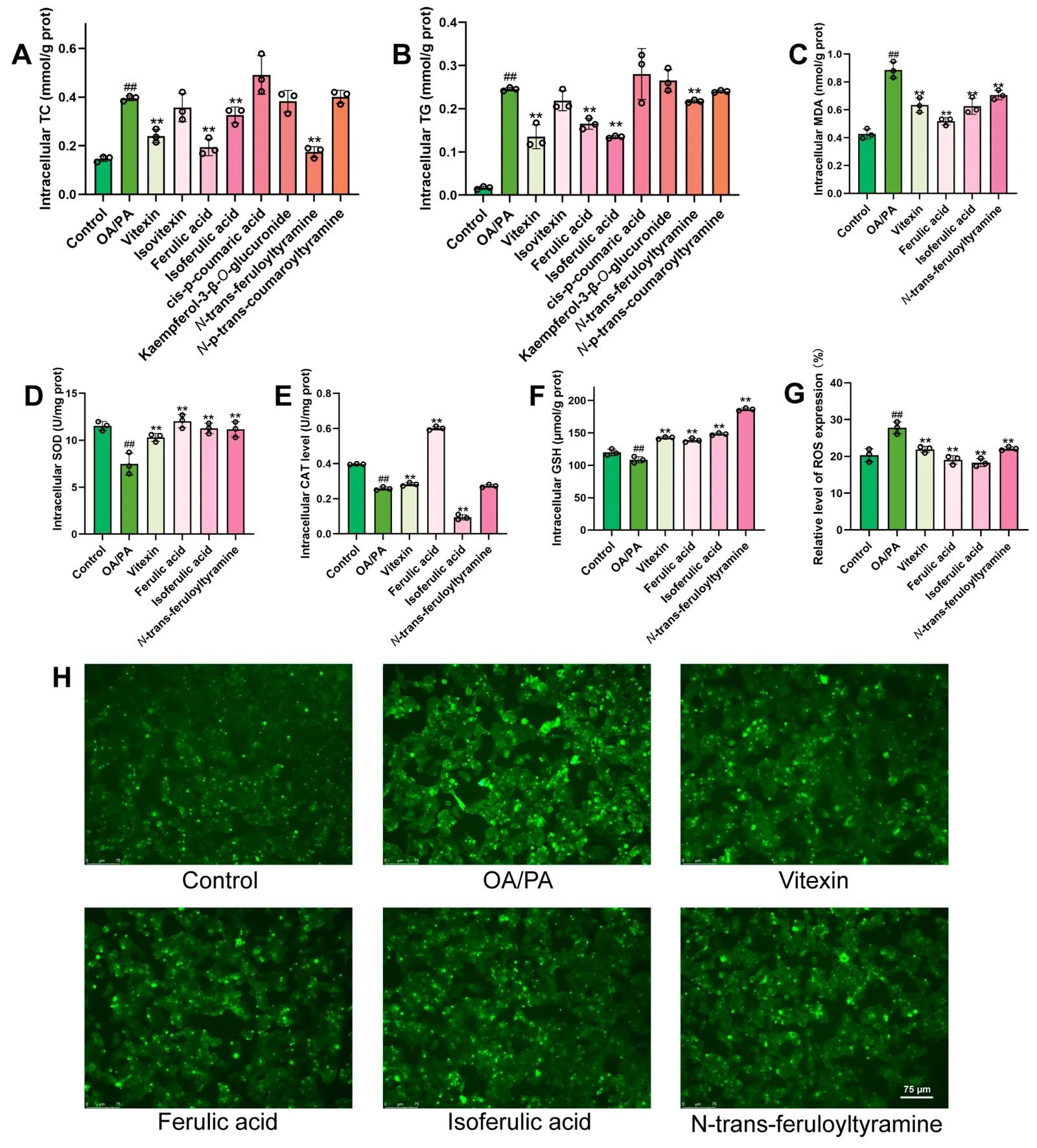
Effects of *M. folium* on lipid accumulation and oxidative stress in HepG2 cells (*n* = 3). (**A**) Intracellular TC. (**B**) Intracellular TG. (**C**) Intracellular MDA. (**D**) Intracellular SOD. (**E**) Intracellular CAT. (**F**) Intracellular GSH. (**G**,**H**) Relative level of ROS expression. All data are represented as mean ± SD. ^##^ *p* < 0.01 as compared to Control; ** *p* < 0.01 as compared to OA/PA.

To identify the *M. folium*-derived compounds with the most pronounced lipid-lowering activity, the eight pharmacokinetically detected constituents were first evaluated in the OA/PA-induced HepG2 lipid accumulation model. As shown in Figure 6A,B, OA/PA exposure markedly increased intracellular TC and TG levels compared with the control group. Treatment with individual *M. folium*-derived compounds produced differential effects on intracellular lipid accumulation. Among the eight compounds tested, vitexin, ferulic acid, isoferulic acid, and *N*-trans-feruloyltyramine significantly decreased both intracellular TC and TG levels compared with the OA/PA model group, whereas the remaining compounds did not produce significant reductions in both lipid parameters. Therefore, these four compounds were selected as candidate active constituents for subsequent validation.

In addition, OA/PA exposure markedly enhanced oxidative stress, characterized by increased ROS and MDA levels, accompanied by decreased activities of antioxidant enzymes, including SOD, CAT, and GSH (Figure 6C–H). These results confirmed the successful establishment of a lipid accumulation model in HepG2 cells.

Treatment with *M. folium* or its representative compounds significantly reduced intracellular TC and TG levels compared with the OA/PA model group. Moreover, drug treatment markedly attenuated OA/PA-induced oxidative stress by decreasing ROS and MDA levels and restoring antioxidant enzyme activities. These findings indicate that *M. folium* effectively alleviates lipid accumulation and oxidative stress in hepatocytes.

#### 2.7.3. Validation of PI3K-Akt Signaling Activation by *M. folium*-Derived Compounds in HepG2 Cells

**Figure 7:**
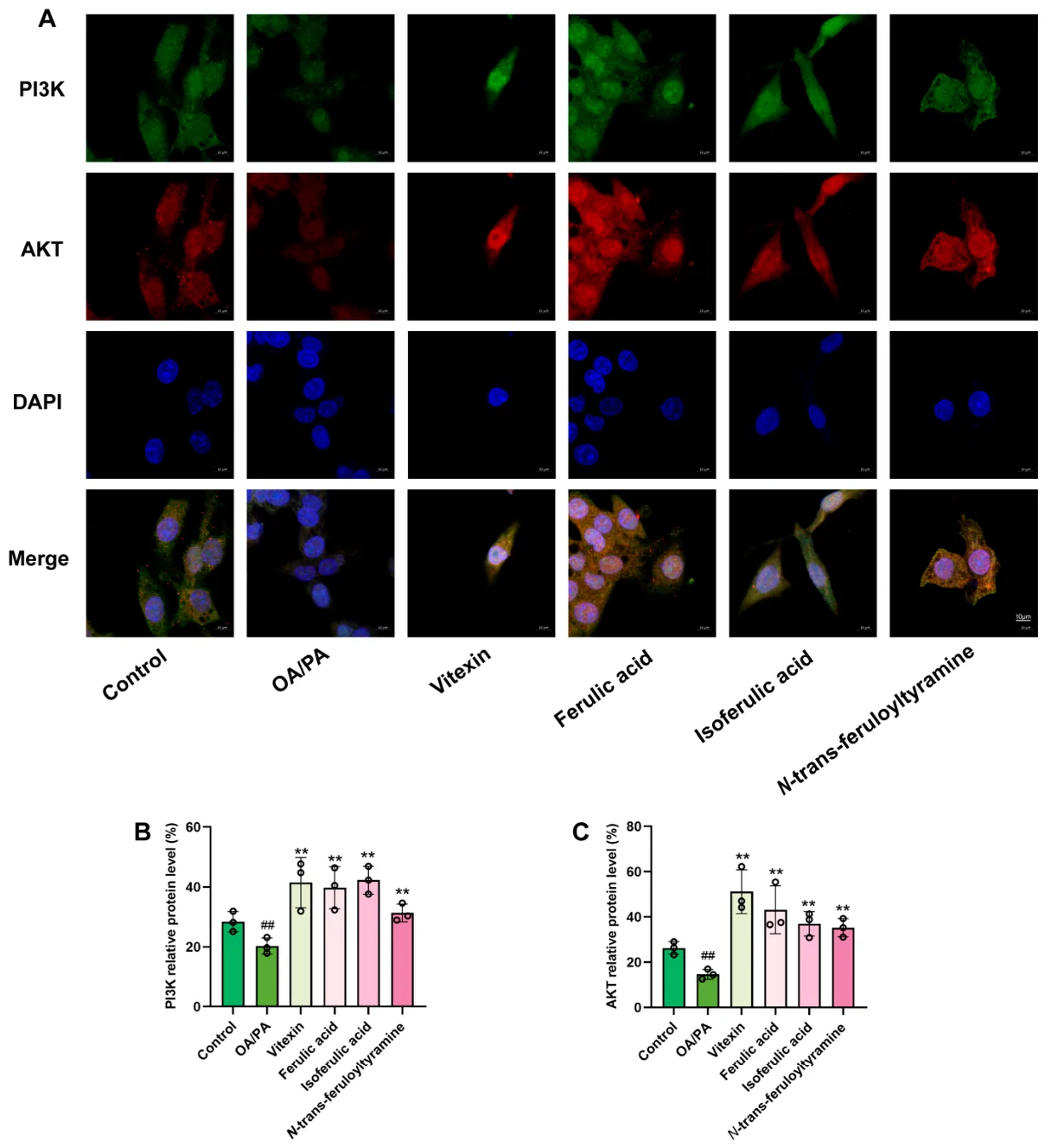
Effects of *M. folium* on PI3K-Akt pathway in HepG2 cells (*n* = 3). (**A**) The expression of PI3K and AKT in HepG2 cells. (**B**) PI3K relative protein level. (**C**) AKT relative protein level. All data are represented as mean ± SD. ^##^ *p* < 0.01 as compared to Control; ** *p* < 0.01 as compared to OA/PA.

Immunofluorescence was performed to further investigate the potential mechanisms underlying the protective effects of *M. folium*. As shown in Figure 7, OA/PA exposure markedly decreased PI3K and AKT fluorescence intensity relative to that observed in the control group, suggesting suppression of the PI3K-Akt signaling pathway.

In contrast, *M. folium* treatment markedly enhanced the expression levels of PI3K and AKT, as indicated by increased fluorescence intensity relative to the OA/PA model group. These results suggest that *M. folium* exerts its lipid-lowering effects through activation of the PI3K-Akt signaling pathway, which is consistent with the network pharmacology predictions.

### 2.8. In Vivo Analysis

#### 2.8.1. Effects of *M. folium* Active Compounds on Serum and Hepatic Lipid Levels

**Figure 8:**
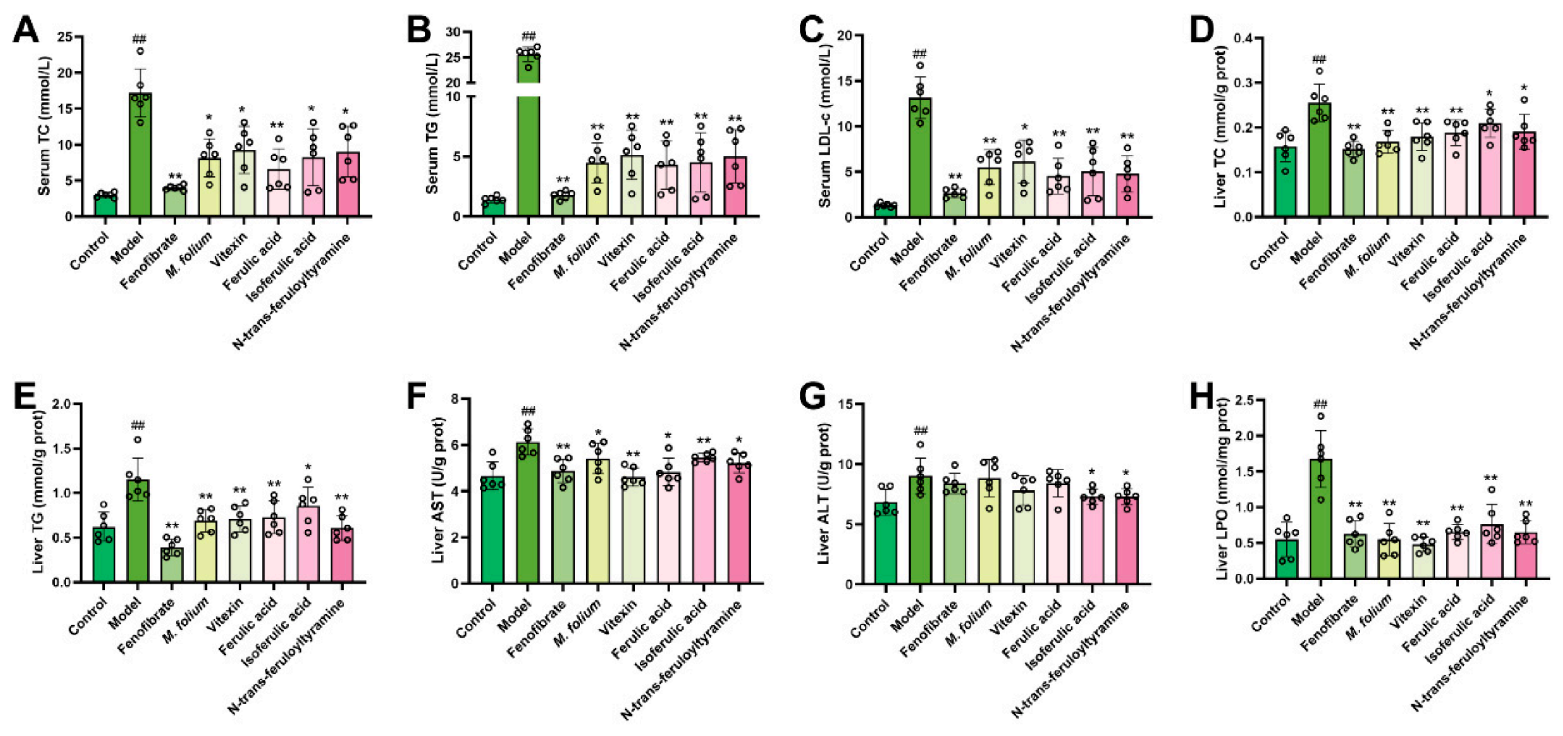
Effects of *M. folium* active compounds on serum and hepatic lipid levels (*n* = 6). (**A**) Serum TC. (**B**) Serum TG. (**C**) Serum LDL-c. (**D**) Liver TC. (**E**) Liver TG. (**F**) Liver AST. (**G**) Liver ALT. (**H**) Liver LPO. All data are represented as mean ± SD. ^##^ *p* < 0.01 as compared to Control; * *p* < 0.05 and ** *p* < 0.01 as compared to Model.

As shown in Figure 8A–H, Triton WR-1339 injection significantly increased serum TC, serum TG, serum LDL-c, liver TC, liver TG, liver AST, liver ALT, and liver LPO levels compared with the control group, confirming the successful establishment of an acute hyperlipidemia model accompanied by hepatic injury and oxidative stress.

Treatment with fenofibrate markedly reduced serum and hepatic lipid levels and improved liver function indices, validating the reliability of the animal model. Compared with the model group, administration of vitexin, ferulic acid, isoferulic acid, and *N*-trans-feruloyltyramine significantly decreased serum TC, TG, and LDL-c levels, as well as hepatic TC and TG levels. In addition, these compounds alleviated liver injury and oxidative stress, as evidenced by reduced AST, ALT, and LPO levels. Among the tested compounds, vitexin and ferulic acid exhibited more pronounced lipid-lowering and hepatoprotective effects, comparable to those observed in the fenofibrate-treated group.

#### 2.8.2. Histopathological Evaluation of Liver Tissues

**Figure 9:**
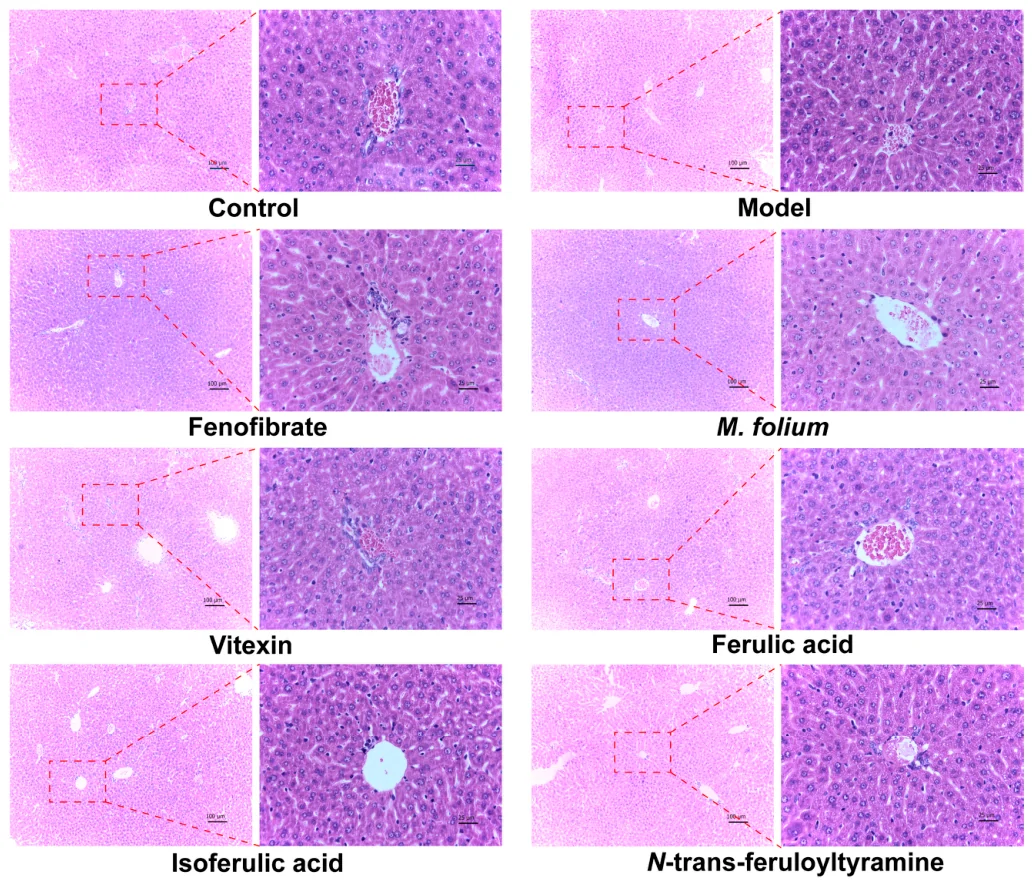
Histopathological evaluation of liver tissues.

Histopathological examination further supported the biochemical findings (Figure 9). Liver sections from control mice displayed normal hepatic architecture with well-organized hepatocytes. In contrast, mice in the model group exhibited obvious hepatic steatosis, characterized by extensive lipid droplet accumulation and hepatocyte swelling.

Treatment with fenofibrate markedly alleviated hepatic steatosis. Similarly, administration of *M. folium*-derived active compounds notably reduced lipid deposition and improved hepatic morphology, with vitexin and ferulic acid showing the most significant protective effects. These results indicate that *M. folium* active compounds effectively attenuate Triton WR-1339-induced hepatic lipid accumulation *in vivo*.

#### 2.8.3. Effects of *M. folium*-Derived Compounds on the PI3K-Akt Signaling Pathway in Liver Tissues

**Figure 10:**
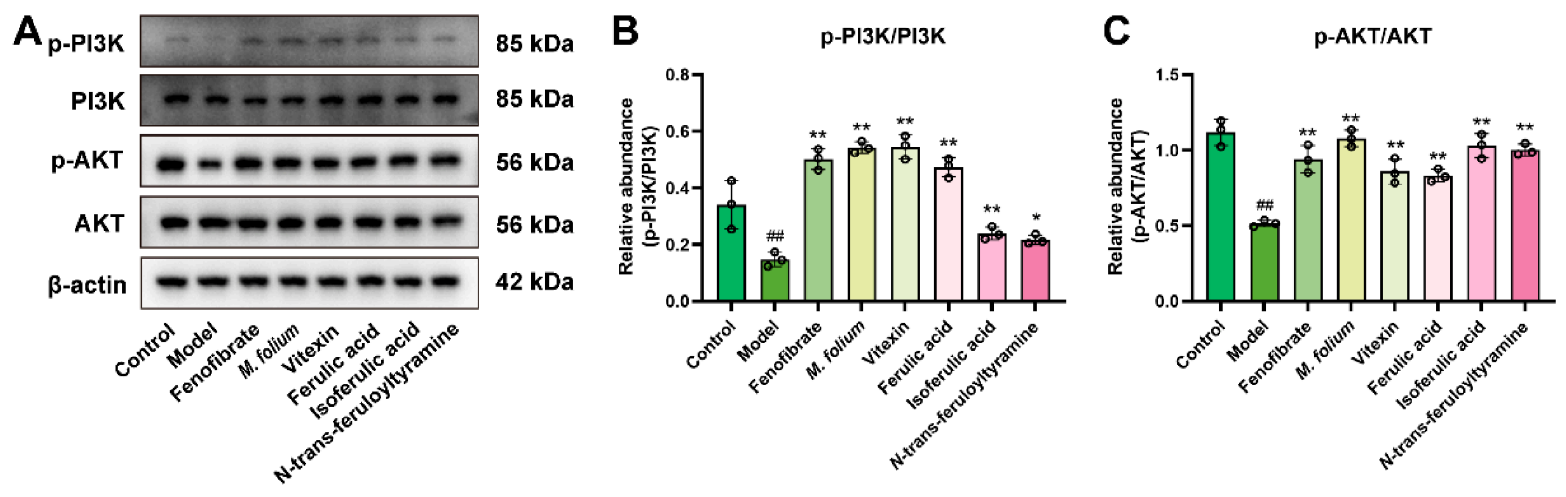
Effects of *M. folium*-derived compounds on the PI3K-Akt pathway (*n* = 3). (**A**) Western blot. (**B**) p-PI3K/PI3K. (**C**) p-AKT/AKT. All data are represented as mean ± SD. ^##^ *p* < 0.01 as compared to Control; * *p* < 0.05 and ** *p* < 0.01 as compared to Model.

To further validate the mechanism predicted by network pharmacology, the phosphorylation status of PI3K and AKT in mouse liver tissues was examined by Western blotting (Figure 10). Compared with the control group, Triton WR-1339 administration significantly decreased the phosphorylation levels of PI3K and AKT, indicating inhibition of the PI3K-Akt pathway during hyperlipidemia development. Treatment with fenofibrate, vitexin, ferulic acid, isoferulic acid, and *N*-trans-feruloyltyramine significantly increased the p-PI3K/PI3K and p-AKT/AKT ratios compared with the model group. These findings indicate that modulation of the PI3K-Akt pathway may be associated with the lipid-lowering effects of *M. folium*-derived active compounds, although the involvement of downstream lipid metabolism-related regulators remains to be further elucidated.

## 3. Discussion

Hyperlipidemia constitutes a central pathological driver of metabolic-associated fatty liver disease and cardiovascular morbidity worldwide. Although statins and fibrates remain first-line therapies, their long-term use is frequently accompanied by adverse effects and limited efficacy in complex metabolic disorders [15]. Consequently, increasing attention has been directed toward natural products with multi-target regulatory properties. In the present study, an integrated strategy combining chemical profiling, pharmacokinetics, network pharmacology, and experimental validation was employed to elucidate the hypolipidemic material basis and mechanisms of *M. folium*.

UPLC fingerprinting coupled with UPLC-QQQ-MS/MS quantitative analysis demonstrated that *M. folium* possesses a chemically stable profile dominated by flavonoids and phenolic acids, particularly flavone C-glycosides. Such compounds are widely reported to exhibit lipid-regulating and antioxidant activities [16,17]. The integration of fingerprint analysis with targeted quantification ensured batch consistency and provided a solid chemical foundation for bioactivity correlation, addressing a common limitation in herbal medicine research where chemical variability compromises pharmacological interpretation.

Pharmacokinetic evaluation revealed that several *M. folium*-derived compounds were absorbed into systemic circulation following oral administration, albeit with distinct exposure and elimination patterns. Flavonoid glycosides such as vitexin and isovitexin displayed relatively higher plasma exposure, whereas phenolic acids such as ferulic acid, isoferulic acid, and cis-p-coumaric acid exhibited faster clearance. These findings emphasize that *in vivo* bioavailability is a critical determinant in active compound screening, as high chemical abundance or strong *in vitro* activity does not necessarily translate into systemic efficacy [17]. Indeed, several studies have demonstrated that many plant polyphenols exhibit limited absorption and rapid metabolism *in vivo*, resulting in significant differences between *in vitro* potency and pharmacological activity after oral administration [18,19]. Incorporating pharmacokinetic behavior therefore strengthened the rational selection of candidate active constituents and improved the reliability of identifying bioactive compounds from complex herbal matrices.

Network pharmacology analysis suggested that *M. folium* exerts hypolipidemic effects through coordinated modulation of multiple targets associated with lipid metabolism, inflammation, and oxidative stress. KEGG enrichment supported the involvement of PI3K-Akt and insulin-related pathways. This pathway is well established in metabolic homeostasis, regulating lipogenesis, insulin sensitivity, and oxidative balance [20,21], providing a mechanistic framework for experimental validation. Although the present findings implicate PI3K-Akt pathway in the lipid-lowering effects of *M. folium*-derived compounds, downstream regulators of lipid metabolism, such as SREBP-1c, ACC, FASN, AMPK, and PPARα, were not examined. Therefore, whether modulation of PI3K-Akt pathway directly affects hepatic lipid synthesis and oxidation remains to be determined. Future studies incorporating these downstream markers and pathway-specific pharmacological or genetic interventions will be required to establish the causal mechanisms underlying the effects of *M. folium*-derived compounds.

Consistent with these predictions, *in vitro* experiments demonstrated that *M. folium* and its representative compounds significantly attenuated OA/PA-induced lipid accumulation and oxidative stress in HepG2 cells. Reductions in intracellular TC and TG levels, together with restoration of antioxidant defenses, indicate that *M. folium* protects hepatocytes against lipid-induced injury. Similar lipid-lowering and antioxidant effects have been reported for flavonoids and phenolic acids in hepatocellular models [22,23]. Moreover, immunofluorescence analysis revealed enhanced PI3K and AKT expression following *M. folium* treatment, lending experimental support to the network-predicted mechanism.

The *in vivo* validation further substantiated these findings. In a Triton WR-1339-induced hyperlipidemia mouse model, *M. folium*-derived active compounds significantly reduced serum and hepatic TC and TG levels and alleviated hepatic steatosis. Vitexin and ferulic acid exhibited particularly pronounced lipid-lowering effects, comparable to the fenofibrate. Previous studies have reported that vitexin and ferulic acid modulate lipid metabolism and inflammatory signaling in dyslipidemic models [10,24], supporting the biological plausibility of the present findings. Histopathological improvement of liver architecture further corroborated the biochemical outcomes. The activation of the PI3K-Akt signaling pathway predicted by network pharmacology was further validated by both immunofluorescence in HepG2 cells and Western blot analysis of liver tissues, providing complementary evidence that *M. folium* exerts its hypolipidemic effects through modulation of the PI3K-Akt pathway.

In addition to vitexin and ferulic acid, isoferulic acid and *N*-trans-feruloyltyramine may also contribute to the lipid-lowering activity of *M. folium*. Isoferulic acid, a structural isomer of ferulic acid, has been reported to exhibit antioxidant and metabolic regulatory properties that may help maintain lipid homeostasis and alleviate oxidative stress associated with dyslipidemia [25,26]. Meanwhile, *N*-trans-feruloyltyramine, a phenylpropanoid amide widely distributed in plants, has attracted attention for its antioxidant and anti-inflammatory activities, and related hydroxycinnamic acid amides have been shown to suppress lipid accumulation and promote hepatic lipid clearance in hepatocyte models [27,28]. Consistent with these findings, histopathological analysis in the present study demonstrated notable improvement in hepatic architecture following treatment with *M. folium*-derived compounds, supporting the biochemical evidence of reduced lipid accumulation. Collectively, these results suggest that multiple constituents of *M. folium* may act synergistically to ameliorate hyperlipidemia through coordinated regulation of lipid metabolism and oxidative stress.

Despite these encouraging findings, several limitations remain. The pharmacokinetic study assessed only short-term plasma exposure after a single dose; longer-term disposition, tissue distribution, and metabolite profiles were not characterized. Rats were used for pharmacokinetic screening and mice for pharmacodynamic validation, and species differences in metabolism and drug disposition therefore limit quantitative extrapolation between models or to humans. In addition, cellular lipid responses and PI3K-Akt pathway were assessed at single predefined time points, precluding conclusions about the onset, persistence, or reversibility of these effects. Finally, the Triton WR-1339 model reflects acute hyperlipidemia rather than chronic metabolic disease. Future studies should integrate pharmacokinetic and pharmacodynamic assessments within the same species, incorporate time-course analyses and human-relevant models, and validate these findings in chronic dietary or genetic models.

Herbal medicines often exert therapeutic effects through synergistic interactions rather than single constituents. Future research should explore compound-compound interactions and optimize formulations to enhance efficacy and safety. Toxicological evaluation and chronic dosing studies will also be essential steps toward translational application.

## 4. Materials and Methods

### 4.1. Chemicals and Reagents

Details of high-purity reference standards are shown in Supplementary Table S1. Triton WR-1339 was obtained from Sigma-Aldrich (Shanghai, China). Fenofibrate (batch number: 27232) was purchased from Abbott Laboratories Limited (Chicago, IL, USA). Total cholesterol (TC, batch number: 20230828), triglycerides (TG, batch number: 20230830) and low-density lipoprotein cholesterol (LDL-c, batch number: 20250328) were purchased from Nanjing Jiancheng Bioengineering Institute (Nanjing, China). Catalase (CAT, batch number: 2407005), glutathione (GSH, batch number: 2406006), malondialdehyde (MDA, batch number: 2406007), superoxide dismutase (SOD, batch number: 2406007), reactive oxygen species (ROS, batch number: 2406006), aspartate aminotransferase (AST, batch number: 2404004), alanine aminotransferase (ALT, batch number: 2405004), and lipid peroxide (LPO, batch number: 2411008) were purchased from Beijing Solarbio Science & Technology Co., Ltd. (Beijing, China). The antibody of p-PI3K (catalog number: AF3241) was purchased from Affinity Biosciences LTD. (Cincinnati, OH, USA). The antibodies of PI3K (catalog number: 60225-1-Ig), p-AKT (catalog number: 66444-1-Ig), AKT (catalog number: 10176-1-AP) were purchased from Proteintech Group, Inc. (Wuhan, China).

### 4.2. Plant Material Collection

Fresh leaves of *Microcos paniculata* L. (named *M. folium*) were collected from Guangdong Province, China (Supplementary Table S2 for information on plant sources), and authenticated by Prof. Fajing Liu, Guangdong Provincial Engineering Technology Research Institute of Traditional Chinese Medicine. A representative batch of *M. folium* (S11) was selected for the pharmacokinetic, cellular, and animal experiments. This batch was selected based on its representative chromatographic profile, showing a fingerprint similarity value of 0.990 relative to the reference fingerprint, together with a chemical composition within the overall range observed among the 21 batches. The same batch was used throughout the pharmacological experiments to minimize variation arising from differences in chemical composition.

### 4.3. Animals and Cell Lines

The male C57BL/6 mice (18 to 22 g, Approval No. 049354, 8 November 2024) and male Sprague–Dawley (SD) rats (180 to 220 g, Approval No. 049314, 23 May 2024) were obtained from the Guangdong Medical Laboratory Animal Center (Guangzhou, China). During the study, rats and mice were housed in a temperature-controlled environment (24 ± 2 °C) with 60 ± 10% humidity under a standard 12-hour light/dark cycle, with ad libitum access to food and water. All experimental procedures involving animals were conducted in full compliance with the Laboratory Animal–Guideline for Ethical Review of Animal Welfare (GB/T 35892-2018) [29] and received approval from the Animal Ethics Committee of the Guangdong Provincial Engineering Technology Institute of Traditional Chinese Medicine. The experimental protocols involving mice adhered to the ARRIVE guidelines.

HepG2 cells (catalog number: ZQ0022) were purchased from Shanghai Zhong Qiao Xin Zhou Biotechnology Co., Ltd. (Shanghai, China).

### 4.4. Plant Material and Extraction

The dried leaves of *M. folium* (S11, 1.0 kg) were pulverized and extracted twice with 50% ethanol (1:10, *w*/*v*) under reflux for 1 h each. The mixed extracts were then filtered, concentrated under reduced pressure, and stored at −20 °C.

### 4.5. UPLC Fingerprint Analysis of M. folium

#### 4.5.1. Preparation of Sample Solutions for UPLC Fingerprint Analysis

The powdered *M. folium* leaves were weighed (0.5 g) and extracted with 50% methanol 25 mL under reflux for 30 min. Following cooling to room temperature, 50% methanol was added to restore the extract to its initial weight. The solution was mixed thoroughly and subsequently filtered through a 0.22 μm microporous membrane.

#### 4.5.2. Preparation of Reference Standard Solutions for UPLC Fingerprint Analysis

Appropriate amounts of reference standards were accurately weighed and dissolved in 50% methanol to prepare solutions containing caffeic acid (206.80 μg/mL), cis-p-coumaric acid (100.00 μg/mL), ferulic acid (334.66 μg/mL), schaftoside (468.44 μg/mL), vitexin (148.00 μg/mL), isovitexin (201.88 μg/mL), violanthin (200.00 μg/mL), kaempferol-3-β-*O*-glucoside (199.14 μg/mL), *N*-trans-feruloyltyramine (117.21 μg/mL), and narcissin (196.25 μg/mL). All solutions were stored at 4 °C prior to analysis.

#### 4.5.3. Chromatographic Conditions for UPLC Fingerprint Analysis

UPLC fingerprint was performed using an Agilent 1290 ultra-high performance liquid chromatography system (Agilent Technologies, Santa Clara, CA, USA). Chromatographic separation was achieved on an Agilent ZORBAX Eclipse XDB-C18 column (2.1 mm × 100 mm, 1.8 μm). The mobile phase consisted of methanol (A) and 0.1% aqueous acetic acid (B), with the following gradient elution program: 0~5 min, 9~20% A; 5~20 min, 20~25% A; 20~40 min, 25~35% A; 40~55 min, 35~38% A; 55~65 min, 38~50% A. The chromatographic analysis was conducted at a flow rate of 0.3 mL/min with the column maintained at 20 °C. Detection was performed at 315 nm, and 1 μL of sample was injected for each analysis.

### 4.6. Quantitative Analysis by UPLC-QQQ-MS/MS

#### 4.6.1. Chromatographic Conditions for Quantitative Analysis

Quantitative determination was conducted using an Agilent 1290 II UPLC system coupled with an Agilent 6495C triple quadrupole mass spectrometer (Agilent Technologies, Santa Clara, CA, USA). Chromatographic separation was carried out using an Agilent Poroshell 120 SB-C18 column (2.1 mm × 100 mm, 2.7 μm) at 45 °C. The eluent comprised methanol (A) and 0.1% formic acid in water (B). The gradient program was set as follows: 12% A from 0 to 7 min; 12~30% A from 7 to 8 min; 30~50% A from 8 to 10 min; 50% A from 10 to 12 min; 50~95% A from 12 to 14 min; and 95% A from 14 to 15 min. The injection volume was 1 μL.

#### 4.6.2. Mass Spectrometric Conditions for Quantitative Analysis

The mass spectrometer operated with an electrospray ionization (ESI), was used for mass spectrometric analysis. Data acquisition was conducted in both positive and negative ionization modes using multiple reaction monitoring (MRM). The main mass spectrometry parameters were set as follows: capillary voltage 3000 V; ion source temperature 500 °C; drying gas temperature 250 °C with a gas flow rate of 11 L/min; nebulizer pressure 30 psi; sheath gas temperature 350 °C with a flow rate of 11 L/min. The optimized MRM transitions and collision energies for each analyte are listed in Supplementary Table S3.

#### 4.6.3. Preparation of Sample Solutions for Quantitative Analysis

Approximately 0.2 g of powdered *M. folium* leaves were accurately weighed and extracted with 50% methanol 20 mL under reflux for 30 min. Following cooling to room temperature, 50% methanol was added to restore the extract to its initial weight. The mixture was thoroughly homogenized and passed through a 0.22 μm membrane filter to prepare the test solution.

#### 4.6.4. Preparation of Reference Standard Solutions for Quantitative Analysis

Reference standards were accurately weighed and dissolved in 50% methanol to prepare individual stock solutions containing schaftoside (468.44 μg/mL), berberine (121.21 μg/mL), vitexin (148.00 μg/mL), isovitexin (201.88 μg/mL), violanthin (200.00 μg/mL), ferulic acid (334.66 μg/mL), cis-p-coumaric acid (100.00 μg/mL), *N*-trans-feruloyltyramine (120.40 μg/mL), *N*-trans-p-coumaroyltyramine (117.21 μg/mL), kaempferol-3-β-*O*-glucoside (199.14 μg/mL), isoferulic acid (257.92 μg/mL), narcissin (196.25 μg/mL), tilianin (207.76 μg/mL), caffeic acid (206.80 μg/mL), epicatechin (111.20 μg/mL), typhaneoside (314.38 μg/mL), isorhamnetin-3-*O*-neohesperidoside (210.26 μg/mL), and rutin (99.60 μg/mL). All standard solutions were stored at 4 °C until analysis.

### 4.7. Pharmacokinetic Study

#### 4.7.1. Animals and Plasma Sample Collection

After acclimatization, 12 rats were randomly divided into a control group and an administration group (*n* = 6). All animals were fasted for 12 h before dosing, with water provided ad libitum. Rats in the administration group were orally administered *M. folium* extract at a human equivalent dose of 2.70 g/kg, the control animals were administered an equivalent volume of distilled water by intragastric gavage.

Blood samples were collected from the retro-orbital venous plexus at 0 min, 2 min, 5 min, 10 min, 20 min, 30 min, 45 min, 1 h, 2 h, 3 h, 4 h, 8 h, 12 h and 24 h after administration. Samples were immediately centrifuged at 3000 rpm for 15 min at 4 °C to obtain plasma. Plasma samples collected at each time point were pooled in equal volumes and stored at −80 °C until analysis.

#### 4.7.2. Plasma Sample Preparation

Plasma samples were individually prepared using a protein precipitation procedure. Plasma samples (100 μL) were deproteinized by adding 400 μL of methanol-acetonitrile (1:1, *v*/*v*) supplemented with the internal standard (IS, 2.011 μg/mL). The mixture was vortexed for 30 s and kept at −20 °C for 1 h to ensure complete protein precipitation.

After centrifugation at 12,000 rpm for 15 min at 4 °C, the supernatant was collected and evaporated to dryness under a gentle nitrogen stream at room temperature. For reconstitution, 100 μL of methanol/water (4:1, *v*/*v*) was added to the residue. After thorough vortexing, the samples were centrifuged at 12,000 rpm for 15 min at 4 °C, and 80 μL of the clear supernatant was collected in an autosampler vial for UPLC-MS/MS determination.

#### 4.7.3. UPLC-MS/MS Conditions for Pharmacokinetic Analysis

Pharmacokinetic analysis was performed using an Agilent 1290II UPLC system coupled with an Agilent 6495C triple quadrupole mass spectrometer (Agilent Technologies, Santa Clara, CA, USA). Chromatographic separation was achieved on an Agilent Extend-C18 column (50 mm × 2.1 mm, 1.8 μm), maintained at 35 °C. Using 0.1% aqueous formic acid and acetonitrile as solvents A and B, respectively, chromatographic separation was achieved with the following gradient: B was maintained at 10% for 0~0.5 min, increased to 16% over 0.5~3 min, held at 16% for 3~6 min, and subsequently raised from 16% to 20% during 6~8 min, from 20% to 38% during 8~9.5 min, and from 38% to 95% during 9.5~10.5 min, followed by a 95% B hold until 13 min. The analysis was conducted at 0.3 mL/min with a 1 μL injection volume.

Mass spectrometric detection was conducted in both positive and negative electrospray ionization modes using MRM. The main parameters were set as follows: capillary voltage 3000 V; ion source temperature 500 °C; drying gas temperature 200 °C with a flow rate of 15 L/min; nebulizer pressure 30 psi; and sheath gas temperature 350 °C with a flow rate of 11 L/min. The optimized MRM transitions and collision energies for all analytes and the internal standard are summarized in Supplementary Table S4.

### 4.8. Network Pharmacology Analysis

#### 4.8.1. Identification of Potential Targets of Active Compounds

The chemical structures of eight representative compounds from *M. folium*, including vitexin, isovitexin, ferulic acid, isoferulic acid, cis-p-coumaric acid, kaempferol-3-β-*O*-glucuronide, *N*-trans-feruloyltyramine, and *N*-trans-p-coumaroyltyramine, were retrieved from the PubChem (Available online: https://pubchem.ncbi.nlm.nih.gov/ (accessed on 7 March 2024)). The simplified molecular input line entry system (SMILES) information of each compound was obtained and used for subsequent target prediction.

Potential protein targets of each compound were predicted using the ChEMBL database, with the species restricted to Homo sapiens. All predicted targets from different compounds were collected and further standardized using the UniProt database to convert protein names into official symbols. After integration and removal of duplicate entries, a comprehensive list of potential targets associated with the active compounds of *M. folium* was obtained.

#### 4.8.2. Collection of Hyperlipidemia-Related Targets

Targets associated with hyperlipidemia were identified by searching the GeneCards, OMIM, and DisGeNET databases using the keyword “hyperlipidemia,” with the species limited to Homo sapiens. Targets identified from the three databases were consolidated, and duplicate records were excluded to obtain the final hyperlipidemia-related target set.

#### 4.8.3. Identification of Overlapping Targets

To explore the potential therapeutic targets through which *M. folium* may exert its lipid-lowering effects, the common targets shared by the compound-related and hyperlipidemia-related target sets were subsequently identified. Venn diagrams were generated using Venn 2.1.1 to visualize the intersection, and the shared targets were selected for further analysis.

#### 4.8.4. Construction of Protein–Protein Interaction (PPI) Network and Identification of Hub Targets

The overlapping targets were imported into the STRING database to construct a PPI network. The organism was set as Homo sapiens, the minimum required interaction score was defined as medium confidence (>0.4), and a stringent false discovery rate (FDR) was applied. The resulting PPI data was used to generate a network representing the interactions among the shared targets, which facilitated the identification of core targets involved in the therapeutic effects of *M. folium* against hyperlipidemia.

#### 4.8.5. GO and KEGG Pathway Enrichment

To elucidate the biological functions and molecular mechanisms underlying the anti-hyperlipidemic effects of *M. folium*, Gene Ontology (GO) functional annotation and Kyoto Encyclopedia of Genes and Genomes (KEGG) pathway enrichment analyses were performed using R software (version 4.0.2). The Bioconductor packages clusterProfiler, org.Hs.eg.db, and DOSE were employed for enrichment analysis.

GO analysis was conducted to evaluate biological processes (BP), cellular components (CC), and molecular functions (MF), while KEGG pathway enrichment was used to identify key signaling pathways associated with the overlapping targets. A significance threshold of *p* < 0.05 and *q* < 0.05 was applied to screen for statistically significant GO and KEGG, thereby revealing the potential biological pathways through which *M. folium* exerts therapeutic effects on hyperlipidemia.

### 4.9. In Vitro Experiments

#### 4.9.1. CCK-8 Assay

The concentrations of the eight compounds used for subsequent cellular experiments were determined based on the CCK-8 cytotoxicity assay.

#### 4.9.2. Measurement of Intracellular Lipid Levels and Oxidative Stress Markers

For subsequent experiments, HepG2 cells were cultured in 6-well plates at 1 × 10^6^ cells/mL and allowed to attach overnight before treatment. Cells were then assigned to the following groups: Control, OA/PA model (oleic acid/palmitic acid, 500/250 μM) [30], and OA/PA plus drug-treated groups. Each treatment was performed in triplicate.

Following 24 h of treatment, the cells were harvested and rinsed with PBS. Cell lysates were prepared by ultrasonic disruption in 500 μL PBS and kept on ice. Intracellular levels of TC, TG, GSH, MDA, SOD, CAT, and ROS were quantified using commercial assay kits according to the manufacturers’ protocols.

#### 4.9.3. Immunofluorescence

Sterilized glass coverslips were placed in 24-well plates, and HepG2 cells were seeded at a density of 2 × 10^4^ cells per well. When cell confluence reached approximately 50%, cells were treated with Control, OA/PA, or OA/PA plus drug for 24 h.

Following PBS washing, the cells were incubated with primary antibodies targeting AKT and PI3K at 4 °C overnight. After 1 h of incubation with fluorescent secondary antibodies in the dark, DAPI was applied for nuclear counterstaining. Coverslips were mounted using anti-fade mounting medium and observed under a fluorescence microscope (Zeiss LSM900, Zeiss, Jena, Germany).

### 4.10. In Vivo Validation

#### 4.10.1. Drug Preparation and Dose Selection

Triton WR-1339 was prepared as a 0.12 g/mL stock solution using physiological saline as the solvent.

Fenofibrate was administered at 26 mg/kg, corresponding to the mouse-equivalent dose calculated from the recommended adult clinical dose (200 mg/day) using body-surface-area conversion. To facilitate a standardized pharmacodynamic comparison among candidate constituents, the same dose of 26 mg/kg was used for vitexin, ferulic acid, isoferulic acid, and *N*-trans-feruloyltyramine. This dose was therefore used as an experimental comparison dose rather than as a compound-specific human-equivalent dose. Also, the *M. folium* group’s dosage is 5.28 g herb/kg [9].

#### 4.10.2. Animal Grouping, Model Establishment, and Drug Administration

Forty-eight mice were acclimatized for 7 days and randomly assigned to eight groups: control, model, fenofibrate, *M. folium*, ferulic acid, vitexin, isoferulic acid, and *N*-trans-feruloyltyramine groups (*n* = 6). Drug-treated mice received the corresponding compounds twice daily for five consecutive days, while the control and model groups administered an equal volume of saline. No animals or data points were excluded from the analysis. Investigators were not blinded to group allocation during treatment and data analysis.

Animal modeling methods and reference literature for drug administration [10] were as follows. Briefly, on the afternoon of day 3, all groups except the control group were intramuscularly injected with Triton WR-1339 (0.1 mL/20 g) to induce an acute metabolic-associated fatty liver disease model. Control mice received an equivalent volume of saline. After model induction, mice were allowed free access to food and water. From the evening of day 4, mice were subjected to a 12-h fast with unrestricted access to water. On day 5, 1 h after the final administration, blood samples were collected via orbital extraction and centrifuged at 3000 rpm for 10 min to obtain serum. Liver tissues were excised, with portions stored at −80 °C for biochemical analysis and others fixed in 4% paraformaldehyde for histopathological examination.

#### 4.10.3. Determination of TC and TG Levels in Serum and Liver

The levels of TC, TG, LDL-c, AST, ALT, and LPO in mouse serum or liver tissues were determined using commercial assay kits according to the manufacturers’ instructions.

#### 4.10.4. Histopathological Analysis

Liver tissues fixed in 4% paraformaldehyde were processed through dehydration, paraffin embedding, sectioning, and hematoxylin-eosin (H&E) staining. Histopathological changes in liver tissues were examined under a light microscope (Leica DM500, Leica, Wetzlar, Germany).

#### 4.10.5. Western Blotting

Liver tissues were subjected to protein extraction using RIPA lysis buffer supplemented with protease and phosphatase inhibitors. Following centrifugation, protein concentrations in the supernatants were assessed by the BCA method. Protein samples were then normalized, separated by SDS-PAGE, and transferred to PVDF membranes. The membranes were blocked and subsequently incubated with primary antibodies at 4 °C overnight. After washing, HRP-labeled secondary antibodies were applied. The signals were captured with a Tanon 5200 imaging system (Shanghai, China), and densitometric analysis was performed using ImageJ version 1.54.

### 4.11. Statistical Analysis

Data are presented as mean ± standard deviation (SD). Statistical analyses were performed using GraphPad Prism 9.0 (San Diego, CA, USA). The normality of continuous variables was assessed using the Shapiro–Wilk test. For normally distributed data, one-way analysis of variance (ANOVA) followed by Dunnett’s multiple-comparisons test was used for comparisons among multiple groups. For comparisons between two groups, an unpaired Student’s *t*-test was applied. Statistical significance was defined as *p* < 0.05. ^#^ *p* < 0.05 and ^##^ *p* < 0.01 indicate comparisons with the control group, whereas * *p* < 0.05 and ** *p* < 0.01 indicate comparisons with the model group.

## 5. Conclusions

These findings indicate that the lipid-lowering activity of *M. folium* involves the synergistic regulation of multiple components, targets, and signaling pathways. By integrating chemical characterization, pharmacokinetics, network pharmacology, and biological validation, vitexin, ferulic acid, isoferulic acid, and *N*-trans-feruloyltyramine were identified as key contributors to *M. folium*’s lipid-lowering activity. This integrative strategy provides a robust framework for elucidating the material basis of traditional herbal medicines and supports the further development of *M. folium* as a promising natural intervention for hyperlipidemia.

## Figures and Tables

**Table 1:** Main pharmacokinetic parameters of eight analytes ($\bar{x}$ ± *s*, *n* = 6).

Component	t_1/2_ (h)	T_max_ (h)	C_max_ (ng/mL)	AUC_0→t_ (h·ng/mL)	AUC_0→∞_ (h·ng/mL)	MRT_0→t_ (h)
Vitexin	3.98 ± 2.96	0.67 ± 0.1	11.56 ± 10.19	15.37 ± 7.96	17.12 ± 9.77	4.46 ± 2.53
Isovitexin	2.56 ± 1.93	0.55 ± 0.30	30.27 ± 13.16	84.68 ± 37.18	89.53 ± 41.16	3.00 ± 1.70
Ferulic acid	1.42 ± 0.78	0.40 ± 0.17	170.17 ± 39.85	147.63 ± 31.13	150.93 ± 30.10	1.12 ± 0.44
Isoferulic acid	5.23 ± 4.11	0.36 ± 0.07	471.97 ± 52.99	538.76 ± 142.07	548.09 ± 137.93	2.18 ± 0.56
Cis-p-coumaric acid	3.19 ± 1.09	0.43 ± 0.17	767.69 ± 100.98	1142.43 ± 362.55	1206.83 ± 411.17	2.22 ± 0.72
Kaempferol-3-β-*O*-glucuronide	3.36 ± 2.24	0.75 ± 0.05	309.44 ± 59.32	729.46 ± 339.14	786.93 ± 443.11	3.24 ± 1.80
*N*-trans-feruloyltyramine	8.47 ± 5.57	0.42 ± 0.19	4.15 ± 1.10	13.82 ± 2.67	25.51 ± 17.44	4.04 ± 0.85
*N*-p-trans-coumaroyltyramine	4.88 ± 1.48	0.22 ± 0.14	82.17 ± 28.39	159.21 ± 48.55	187.73 ± 61.52	3.81 ± 1.47

## Data Availability

The original contributions presented in the study are included in the article and Supplementary Material, further inquiries can be directed to the corresponding author. The raw data supporting the conclusions of this article will be made available by the authors on request.

## Supplementary Materials

The following supporting information can be downloaded at: https://www.mdpi.com/article/10.3390/ph19091349/s1, Figure S1: Process of administration of *M. folium* and its active components and hyperlipemia modeling; Figure S2: The structural formulas of eight compounds; Table S1: Information on the reference standards; Table S2. Information on *M. folium*; Table S3: Parameters of mass spectrometry of the 18 analytes; Table S4: Parameters of mass spectrometry of the eight analytes and IS; Table S5: Similarity evaluation of *M. folium* fingerprints for 21 batches; Table S6: Linear ranges, regression equations, correlation coefficients, quantification limits of 18 components; Table S7: Quantitative analysis by UPLC-QQQ-MS/MS for 21 batches *M. folium* (*n* = 2); Table S8: Regression equations, linear ranges, correlation coefficients and LLOQs of analytes.

## References

[1] Das M., Kumar G.S. (2021). Potential role of mycosterols in hyperlipidemia—A review. Steroids.

[2] Tatley M., Savage R. (2007). Psychiatric adverse reactions with statins, fibrates and ezetimibe: Implications for the use of lipid-lowering agents. Drug Saf..

[3] Song Z., Bu S., Sang S., Li J., Zhang X., Song X., Ran Y. (2025). The Active Components of Traditional Chinese Medicines Regulate the Multi-Target Signaling Pathways of Metabolic Dysfunction-Associated Fatty Liver Disease. Drug Des. Devel Ther..

[4] Guo X., Liu C., Dong Z., Luo G., Li Q., Huang M. (2024). Flavonoids from Rhododendron nivale Hook. f ameliorate alcohol-associated liver disease via activating the PPARα signaling pathway. Phytomedicine.

[5] Gao Y., Zhou Q., Wang H., Xin G., Wang T., Zhang K., Yu X., Wen A., Wu Q., Li X. (2024). Isoxanthohumol improves hepatic lipid metabolism via regulating the AMPK/PPARα and PI3K/AKT signaling pathways in hyperlipidemic mice. Food Sci. Nutr..

[6] Lombardo G.E., Navarra M., Cremonini E. (2024). A flavonoid-rich extract of bergamot juice improves high-fat diet-induced intestinal permeability and associated hepatic damage in mice. Food Funct..

[7] Wang C., Yang F., Zeng W., Chen X., Qiu Z., Wang Q., Meng Y., Zheng G., Hu J. (2024). Vine tea total flavonoids activate the AMPK/mTOR pathway to amelioration hepatic steatosis in mice fed a high-fat diet. J. Food Sci..

[8] Qiu L., Gao C., Wang H., Ren Y., Li J., Li M., Du X., Li W., Zhang J. (2023). Effects of dietary polyphenol curcumin supplementation on metabolic, inflammatory, and oxidative stress indices in patients with metabolic syndrome: A systematic review and meta-analysis of randomized controlled trials. Front Endocrinol..

[9] Xiao G., Wu G., Liu Y., Chen W., Zeng Z., Li S., Li Y., Bi X. (2025). Mechanisms of Microctis Folium in Hyperlipidemia: Integrating Serum Pharmacochemistry, Network Pharmacology, and Transcriptomics. Drug Des. Devel Ther..

[10] Zeng Z., Xiao G., Liu Y., Wu M., Wei X., Xie C., Wu G., Jia D., Li Y., Li S. (2024). Metabolomics and network pharmacology reveal partial insights into the hypolipidemic mechanisms of ferulic acid in a dyslipidemia mouse model. Front Pharmacol..

[11] Li S., Liang T., Zhang Y., Huang K., Yang S., Lv H., Chen Y., Zhang C., Guan X. (2021). Vitexin alleviates high-fat diet induced brain oxidative stress and inflammation via anti-oxidant, anti-inflammatory and gut microbiota modulating properties. Free Radic. Biol. Med..

[12] Sharma U., Singh T., Agrawal V. (2025). Phytochemical Analysis, Isolation, and Characterization of Gentiopicroside from Gentiana kurroo and Cytotoxicity of Biosynthesized Silver Nanoparticles Against HeLa Cells. Appl. Biochem Biotechnol..

[13] Hu L., Luo J., Wen G., Sun L., Liu W., Hu H., Li J., Wang L., Su W., Lin L. (2023). Identification of the active compounds in the Yi-Fei-San-Jie formula using a comprehensive strategy based on cell extraction/UPLC-MS/MS, network pharmacology, and molecular biology techniques. Phytomedicine.

[14] Chen S., Qi H., Zhu C., Zhao Y., Jiao B., Tan Y., Yang Y., Wang T., Hou Y., Dai B. (2025). Quality and composition control of complex TCM preparations through a novel “Herbs-*in vivo* Compounds-Targets-Pathways” network methodology: The case of Lianhuaqingwen capsules. Pharmacol. Res..

[15] Grundy S.M., Stone N.J., Bailey A.L., Beam C., Birtcher K.K., Blumenthal R.S., Braun L.T., de Ferranti S., Faiella-Tommasino J., Forman D.E. (2019). 2018 AHA/ACC/AACVPR/AAPA/ABC/ACPM/ADA/AGS/APhA/ASPC/NLA/PCNA Guideline on the Management of Blood Cholesterol: A Report of the American College of Cardiology/American Heart Association Task Force on Clinical Practice Guidelines. Circulation.

[16] Panche A.N., Diwan A.D., Chandra S.R. (2016). Flavonoids: An overview. J. Nutr. Sci..

[17] Pérez-Jiménez J., Neveu V., Vos F., Scalbert A. (2010). Identification of the 100 richest dietary sources of polyphenols: An application of the Phenol-Explorer database. Eur. J. Clin. Nutr..

[18] Chen L., Cao H., Huang Q., Xiao J., Teng H. (2022). Absorption, metabolism and bioavailability of flavonoids: A review. Crit. Rev. Food Sci. Nutr..

[19] Di Lorenzo C., Colombo F., Biella S., Stockley C., Restani P. (2021). Polyphenols and Human Health: The Role of Bioavailability. Nutrients.

[20] Cheng C., Liu X.H., He J., Gao J., Zhou J.T., Fan J.N., Jin X., Zhang J., Chang L., Xiong Z. (2022). Apolipoprotein A4 Restricts Diet-Induced Hepatic Steatosis via SREBF1-Mediated Lipogenesis and Enhances IRS-PI3K-Akt Signaling. Mol. Nutr. Food Res..

[21] Manning B.D., Toker A. (2017). AKT/PKB Signaling: Navigating the Network. Cell.

[22] Samec M., Mazurakova A., Lucansky V., Koklesova L., Pecova R., Pec M., Golubnitschaja O., Al-Ishaq R.K., Caprnda M., Gaspar L. (2023). Flavonoids attenuate cancer metabolism by modulating Lipid metabolism, amino acids, ketone bodies and redox state mediated by Nrf2. Eur. J. Pharmacol..

[23] Li J., Lu X., Zou X., Ye B.C. (2024). Recent Advances in Microbial Metabolic Engineering for Production of Natural Phenolic Acids. J. Agric. Food Chem..

[24] Liu B., Yao Z., Song L., Sun C., Shen C., Cheng F., Cheng Z., Zhang R., Liu R. (2025). Vitexin alleviates lipid metabolism disorders and hepatic injury in obese mice through the PI3K/AKT/mTOR/SREBP-1c pathway. Eur. J. Med. Chem..

[25] Li P.L., Zhai X.X., Wang J., Zhu X., Zhao L., You S., Sang C.Y., Yang J.L. (2023). Two Ferulic Acid Derivatives Inhibit Neuroinflammatory Response in Human HMC3 Microglial Cells via NF-κB Signaling Pathway. Molecules.

[26] Kowalska-Baron A. (2025). Theoretical Insight into Antioxidant Mechanisms of Trans-Isoferulic Acid in Aqueous Medium at Different pH. Int. J. Mol. Sci..

[27] Zhao B., Micklefield J., Wang Y., Wang F. (2025). Biocatalytic Synthesis of N-trans-feruloyltyramine Using an Amide Bond Synthetase with an ATP Recycling. Appl. Biochem Biotechnol..

[28] Shlisky J., Kalgaonkar S., Bloszies C.S., van Kinken J.W., Ferruzzi M.G. (2026). Bioavailability and Metabolism of N-Trans Caffeoyltyramine and N-Trans Feruloyltyramine—A Narrative Review. J. Diet. Suppl..

[29] (2018). Laboratory Animal—Guideline for Ethical Review of Animal Welfare.

[30] Killingsworth Z.K., Misare K.R., Ryan A.S., Ampolini E.A., Mendenhall T.T., Engevik M.A., Hartman J.H. (2024). Subcellular expression of CYP2E1 in HepG2 cells impacts response to free oleic and palmitic acid. Curr. Res. Toxicol..

