# Supplementary material for: Identification of Hypolipidemic Constituents from Microctis folium Using an Integrated Strategy of Chemical Profiling, Pharmacokinetics, Network Pharmacology and Experimental Validation
Source: Pharmaceuticals (Basel). 2026 Aug 26;19(9):1349. doi: 10.3390/ph19091349 (PMC13610286; doi:10.3390/ph19091349)
Supplement: Supplementary file 1 [file pharmaceuticals-19-01349-s001.zip › pharmaceuticals-4494122-supplementary.pdf]

Supplementary Information

Identification of Hypolipidemic Constituents from *Microctis folium* Using an Integrated Strategy of Chemical Profiling, Pharmacokinetics, Network Pharmacology and Experimental Validation

Zhihao Zeng [1,2], Yanchang Liu [3], Xiaoli Bi [1,2], Wanchun Chen [3], Yunhui Ouyang [3], Jingnian Zhang [1,2], Weitao Chen [1,2] and Guanlin Xiao [1,2,*]

1 Guangdong Provincial Engineering Technology Research Institute of Traditional Chinese Medicine, Guangzhou 510095, China; zengzhihao129@126.com (Z.Z.); zyfxyjs@gzucm.edu.cn (X.B.); zjn9110@163.com (J.Z.); chenwt009@126.com (W.C.)

2 Guangdong Provincial Key Laboratory of Research and Development in Traditional Chinese Medicine, Guangzhou 510095, China

3 The Fifth Clinical College of Guangzhou University of Chinese Medicine, Guangzhou 510095, China; 1022905399@qq.com (Y.L.); zxzxas202012@163.com (W.C.); 2907228835@qq.com (Y.O.)

* Correspondence: 164669079@qq.com

Table S1 Information on the reference standards

No.	Reference substances	Lot number	Purity	Supplier company
1	Schaftoside	111912-201703	95.6%	National Institutes for Food and Drug Control
2	Berberine	110713-202515	85.9%	National Institutes for Food and Drug Control
3	Isorhamnetin 3-*O*-neohesperidoside	111571-201205	93.2%	National Institutes for Food and Drug Control
4	Narcissoside	111997-201501	93.1%	National Institutes for Food and Drug Control
5	Caffeic acid	110885-201703	99.7%	National Institutes for Food and Drug Control
6	Epicatechin	110878-201703	99.7%	National Institutes for Food and Drug Control
7	Ferulic acid	110773-201915	99.4%	National Institutes for Food and Drug Control
8	Isoferulin acid	111698-201904	99.3%	National Institutes for Food and Drug Control
9	Rutin	100080-202012	92.2%	National Institutes for Food and Drug Control
10	Vitexin	M-023-120429	>98.0%	Chengdu Herbpurify Co. Ltd.
11	Isovitexin	Y-116-120630	>98.0%	Chengdu Herbpurify Co. Ltd.
12	Typhaneoside	X-020-120305	>98.0%	Chengdu Herbpurify Co. Ltd.
13	*N*-trans-feruloyltyramine	RFS-F03302108016	>98.0%	Chengdu Herbpurify Co. Ltd.
14	Tiliroside	PRF9101501	>98.0%	Chengdu Herbpurify Co. Ltd.
15	Kaempferol-3-β-*O*-glucuronide	DSTDS017501	>98.0%	Chengdu Biopurify Phytochemicals Ltd.
16	Sulfamethoxazole	DSTD200226-220	>98.0%	Chengdu Biopurify Phytochemicals Ltd.
17	Cis-p-coumaric acid	AF20041951	>98.0%	Chengdu Alfa Biotechnology Co. Ltd.
18	Violanthin	AF802612	>98.0%	Chengdu Alfa Biotechnology Co. Ltd.
19	*N*-p-trans-coumaroyltyramine	AF22032709	>98.0%	Chengdu Alfa Biotechnology Co. Ltd.

Table S2. Information on *M. folium*

No.	Batch No.	Collection source	Collection date
S1	20230115	Yangjiang, Guangdong, China	January 2023
S2	20230118	Yangjiang, Guangdong, China	January 2023
S3	20230122	Yangchun, Guangdong, China	January 2023
S4	20230216	Maoming, Guangdong, China	February 2023
S5	20230220	Maoming, Guangdong, China	February 2023
S6	20230318	Huazhou, Guangdong, China	March 2023
S7	20230325	Dianbai, Guangdong, China	March 2023
S8	20230419	Zhanjiang, Guangdong, China	April 2023
S9	20230423	Zhanjiang, Guangdong, China	April 2023
S10	20230514	Maoming, Guangdong, China	May 2023
S11	20230518	Jiangmen, Guangdong, China	May 2023
S12	20230716	Taishan, Guangdong, China	July 2023
S13	20230815	Zhongshan, Guangdong, China	August 2023
S14	20230821	Guangzhou, Guangdong, China	August 2023
S15	20231117	Zhaoqing, Guangdong, China	November 2023
S16	20231123	Yunfu, Guangdong, China	November 2023
S17	20240113	Huizhou, Guangdong, China	January 2024
S18	20240127	Taishan, Guangdong, China	January 2024
S19	20240217	Taishan, Guangdong, China	February 2024
S20	20240228	Zhanjiang, Guangdong, China	February 2024
S21	20240316	Zhanjiang, Guangdong, China	March 2024

Table S3. Parameters of mass spectrometry of the 18 analytes.

No.	Compound	ESI mode	Precursor Ion (*m/z*)	Product Ion (*m/z*)	CE (V)
1	Berberine	ESI+	336.1	292.1	36
				321.1	24
2	Caffeic acid	ESI-	179.0	135.0	13
				134.0	13
3	Cis-p-coumaric acid	ESI-	163.0	119.1	16
				92.9	36
4	Epicatechin	ESI-	289.1	245.1	15
				109.0	25
5	Ferulic acid	ESI-	193.1	177.8	12
				134.2	16

6	Isoferulin acid	ESI-	461.0	178.1<br>134.1	8<br>16
7	Isorhamnetin-3-*O*-neohesperidoside	ESI-	623.2	314.0<br>271.0	35<br>60
8	Isovitexin	ESI-	431.1	311.0<br>283.1	24<br>38
9	Kaempfenol-3-β-*O*-glucuronide	ESI-	461.0	285.0<br>228.0	20<br>44
10	Narcissoside	ESI-	623.1	314.9<br>313.9	32<br>40
11	*N*-p-trans-coumaroyltyamine	ESI+	314.1	177.1<br>145.1	16<br>28
12	*N*-trans-feruloyltyamine	ESI+	284.1	147.1<br>121.2	16<br>28
13	Rutin	ESI-	609.0	301.1<br>300.1	32<br>40
14	Schaftoside	ESI-	563.1	443.2<br>382.9	32<br>40
15	Tiliroside	ESI-	592.9	285.1<br>284.0	32<br>38
16	Typhaneoside	ESI-	769.2	314.0<br>299.0	45<br>65
17	Violanthin	ESI-	577.1	382.9<br>353.1	40<br>40
18	Vitexin	ESI-	431.1	311.1<br>283.0	24<br>36

Table S4 Parameters of mass spectrometry of the eight analytes and IS.

No.	Compound	ESI mode	Precursor Ion (*m/z*)	Product Ion (*m/z*)	CE (V)
1	Cis-p-coumaric acid	ESI-	163.0	119.1<br>92.9	16<br>36
2	Ferulic acid	ESI-	193.1	177.8<br>134.2	12<br>16
3	Isoferulin acid	ESI-	461.0	178.1<br>134.1	8<br>16
4	Isovitexin	ESI-	431.1	311.0<br>283.1	24<br>38
5	Kaempfenol-3-β-*O*-glucuronide	ESI-	461.0	285.0<br>228.0	20<br>44

6	*N*-p-trans-coumaroyltyamine	ESI+	314.1	177.1	16
				145.1	28
7	*N*-trans-feruloyltyamine	ESI+	284.1	147.1	16
				121.2	28
8	Vitexin	ESI-	431.1	311.1	24
				283.0	36
9	Sulfamethoxazole（IS）	ESI-	251.9	156.0	12

Table S5 Similarity evaluation of *M. folium* fingerprints for 21 batches.

S1	S2	S3	S4	S5	S6	S7	S8	S9	S10	S11	S12	S13	S14	S15	S16	S17	S18	S19	S20	S21	Reference
1.000	0.940	0.971	0.950	0.947	0.976	0.944	0.925	0.855	0.925	0.980	0.989	0.965	0.927	0.913	0.982	0.974	0.967	0.876	0.799	0.983	0.983
0.940	1.000	0.938	0.902	0.916	0.963	0.957	0.896	0.931	0.923	0.951	0.949	0.934	0.885	0.919	0.958	0.928	0.936	0.858	0.805	0.979	0.963
0.971	0.938	1.000	0.928	0.936	0.984	0.985	0.873	0.909	0.889	0.962	0.975	0.999	0.88	0.919	0.947	0.988	0.991	0.934	0.869	0.957	0.980
0.950	0.902	0.928	1.000	0.967	0.943	0.925	0.790	0.826	0.785	0.885	0.969	0.93	0.783	0.879	0.917	0.941	0.945	0.779	0.665	0.937	0.907
0.947	0.916	0.936	0.967	1.000	0.933	0.928	0.785	0.832	0.802	0.908	0.978	0.942	0.779	0.900	0.921	0.954	0.931	0.804	0.681	0.943	0.915
0.976	0.963	0.984	0.943	0.933	1.000	0.982	0.887	0.940	0.904	0.957	0.978	0.977	0.891	0.953	0.965	0.971	0.993	0.895	0.838	0.981	0.983
0.944	0.957	0.985	0.925	0.928	0.982	1.000	0.842	0.942	0.865	0.937	0.958	0.986	0.841	0.925	0.929	0.973	0.986	0.916	0.857	0.949	0.965
0.925	0.896	0.873	0.790	0.785	0.887	0.842	1.000	0.772	0.991	0.961	0.871	0.857	0.997	0.792	0.952	0.873	0.859	0.863	0.847	0.919	0.947
0.855	0.931	0.909	0.826	0.832	0.94	0.942	0.772	1.000	0.825	0.857	0.879	0.901	0.778	0.950	0.859	0.866	0.924	0.832	0.814	0.908	0.902
0.925	0.923	0.889	0.785	0.802	0.904	0.865	0.991	0.825	1.000	0.969	0.882	0.874	0.990	0.835	0.957	0.881	0.873	0.881	0.869	0.936	0.959
0.980	0.951	0.962	0.885	0.908	0.957	0.937	0.961	0.857	0.969	1.000	0.962	0.955	0.961	0.889	0.978	0.959	0.942	0.909	0.856	0.973	0.990
0.989	0.949	0.975	0.969	0.978	0.978	0.958	0.871	0.879	0.882	0.962	1.000	0.973	0.871	0.936	0.961	0.973	0.970	0.852	0.760	0.983	0.968
0.965	0.934	0.999	0.93	0.942	0.977	0.986	0.857	0.901	0.874	0.955	0.973	1.000	0.863	0.910	0.939	0.989	0.987	0.932	0.861	0.950	0.973
0.927	0.885	0.88	0.783	0.779	0.891	0.841	0.997	0.778	0.990	0.961	0.871	0.863	1.000	0.800	0.950	0.875	0.866	0.873	0.861	0.916	0.949
0.913	0.919	0.919	0.879	0.900	0.953	0.925	0.792	0.95	0.835	0.889	0.936	0.910	0.800	1.000	0.890	0.891	0.932	0.785	0.733	0.944	0.914
0.982	0.958	0.947	0.917	0.921	0.965	0.929	0.952	0.859	0.957	0.978	0.961	0.939	0.950	0.890	1.000	0.959	0.948	0.893	0.832	0.981	0.985
0.974	0.928	0.988	0.941	0.954	0.971	0.973	0.873	0.866	0.881	0.959	0.973	0.989	0.875	0.891	0.959	1.000	0.983	0.932	0.851	0.949	0.974
0.967	0.936	0.991	0.945	0.931	0.993	0.986	0.859	0.924	0.873	0.942	0.97	0.987	0.866	0.932	0.948	0.983	1.000	0.916	0.853	0.957	0.973
0.876	0.858	0.934	0.779	0.804	0.895	0.916	0.863	0.832	0.881	0.909	0.852	0.932	0.873	0.785	0.893	0.932	0.916	1.000	0.977	0.854	0.933
0.799	0.805	0.869	0.665	0.681	0.838	0.857	0.847	0.814	0.869	0.856	0.760	0.861	0.861	0.733	0.832	0.851	0.853	0.977	1.000	0.788	0.885
0.983	0.979	0.957	0.937	0.943	0.981	0.949	0.919	0.908	0.936	0.973	0.983	0.950	0.916	0.944	0.981	0.949	0.957	0.854	0.788	1.000	0.981

Table S6 Linear ranges, regression equations, correlation coefficients, quantification limits of 18 components.

No.	Compounds	Regression equation	Range (ng/mL)	*R*	LLOQ (ng/mL)
1	Berberine	Y=3718.410X+992.170	0.012~121.210	0.998	0.024
2	Caffeic acid	Y=92.488X+3196.989	2.068~2068.000	0.999	0.041
3	Cis-p-coumaric acid	Y=67.807X+28607.532	1.000~1000.000	0.998	0.020
4	Epicatechin	Y=22.407X+6276.769	111.200~35584.000	0.999	0.111
5	Ferulic acid	Y=21.016X+1308.772	104.580~1673.280	1.000	0.205
6	Isoferulin acid	Y=1.185X-415.286	322.400~41267.200	0.999	0.322
7	Isorhamnetin-3-*O*-neohesperidoside	Y=63.793X+13493.205	65.706~8410.368	0.999	0.066
8	Isovitexin	Y=81.336X+14007.920	252.350~16150.400	0.999	0.504
9	Kaempfenol-3-β-*O*-glucuronide	Y=43.706X+485.329	0.012~124.460	0.998	0.024
10	Narcissoside	Y=7.995X+1590.879	122.659~15700.380	0.999	0.245
11	*N*-p-trans-coumaroyltyamine	Y=1077.021X+577308.143	1.465~146.510	0.996	0.293
12	*N*-trans-feruloyltyamine	Y=1420.386X+538000.889	0.310~150.500	0.997	0.062
13	Rutin	Y=45.748X-8125.029	124.500~7968.000	0.999	0.125
14	Schaftoside	Y=23.173X+3905.399	2.928~292.755	0.999	0.293
15	Tiliroside	Y=48.708X+9904.386	129.850~2077.600	0.995	0.026
16	Typhaneoside	Y=25.304X+3559.870	0.786~392.980	1.000	0.157
17	Violanthin	Y=24.969X+3659.782	125.000~16000.000	1.000	0.250
18	Vitexin	Y=95.438X-238.899	185.000~23680.000	1.000	0.185

Table S7 Quantitative analysis by UPLC-QQQ-MS/MS for 21 batches *M. folium* (*n* = 2).

Compounds	S1	S2	S3	S4	S5	S6	S7	S8	S9	S10	S11	S12	S13	S14	S15	S16	S17	S18	S19	S20	S21
Berberine (μg/g)	0.154	0.147	0.069	0.107	0.077	0.031	0.163	0.049	0.028	0.358	0.033	0.065	0.018	0.016	0.105	0.081	0.020	0.016	0.018	0.016	0.026
Caffeic acid (μg/g)	39.119	20.450	21.054	32.470	25.959	23.351	7.470	87.447	18.590	68.190	51.499	30.412	14.523	55.973	102.167	1.756	17.061	33.665	16.318	59.167	21.265
Cis-p-coumaric acid (μg/g)	20.593	55.005	31.832	43.126	6.912	29.569	38.980	88.884	90.933	68.283	33.926	0.305	12.209	73.926	4.453	25.782	7.578	79.230	42.919	127.393	15.511
Epicatechin (μg/g)	1221.215	377.078	342.940	839.182	390.565	524.798	166.326	881.377	135.901	784.929	1564.158	1154.120	341.500	551.987	489.057	291.737	150.857	1069.511	1057.657	264.740	2756.980
Ferulic acid (μg/g)	119.254	70.507	77.694	93.223	73.906	90.914	80.598	144.189	107.952	138.514	124.908	100.473	59.263	128.180	154.603	75.306	58.636	156.428	83.348	113.552	93.675
Isoferulin acid (μg/g)	253.958	1385.419	1532.708	167.918	239.442	125.319	184.885	212.118	2063.612	2625.249	171.586	216.120	96.000	2391.355	208.245	154.556	107.180	237.254	214.384	161.803	190.720
Isorhamnetin-3-*O*-neohesperidoside (μg/g)	561.142	304.460	13.182	10.072	77.426	0.324	333.376	625.839	263.261	537.790	564.232	57.296	383.797	587.896	348.550	61.683	304.221	491.170	450.000	419.826	441.034
Isovitexin (μg/g)	1088.453	486.623	540.132	354.725	288.351	485.647	322.644	2349.958	353.870	2237.536	1462.261	745.073	367.166	2194.429	576.863	692.074	268.365	609.046	889.345	1237.870	877.733
Kaempfenol-3-β-*O*-glucuronide (μg/g)	0.003	0.002	0.003	0.002	0.002	0.002	0.003	0.033	0.005	0.004	0.002	0.012	0.253	0.004	0.005	0.001	0.001	0.579	0.001	0.001	0.002
Narcissoside (μg/g)	10129.298	5797.909	8149.749	8933.855	7649.397	6436.139	6368.521	11375.431	5179.642	9928.814	10373.353	9527.848	7129.617	10748.127	6549.220	6667.863	5788.115	9168.382	8364.555	8189.030	8204.540
N-p-trans-coumaroyltyamine (μg/g)	3.094	2.489	2.613	2.241	5.787	3.690	3.119	1.964	1.514	2.255	1.880	4.681	3.551	1.975	4.647	4.391	3.736	2.172	2.158	1.742	2.650
N-trans-feruloyltyamine (μg/g)	0.457	0.471	0.736	0.582	1.206	0.813	0.639	0.156	0.278	0.193	0.344	0.829	0.819	0.230	0.651	1.530	0.913	0.402	0.183	0.084	0.280
Rutin (μg/g)	579.558	113.721	162.623	378.041	700.693	183.768	406.635	510.273	79.758	456.195	452.337	550.495	147.575	521.607	535.955	115.222	240.625	215.769	435.424	122.029	210.742

Schaftoside (μg/g)	6.558	5.137	7.199	3.873	3.455	4.211	4.468	15.308	6.813	13.522	7.142	5.363	6.519	12.307	4.002	5.781	3.620	7.428	19.424	19.221	6.436
Tiliroside (μg/g)	51.348	76.691	72.094	47.287	30.295	43.473	8.887	162.559	56.544	177.649	104.530	47.604	33.322	162.067	63.504	130.915	53.392	79.743	201.983	324.148	67.444
Typhaneoside (μg/g)	34.984	5.037	229.188	234.693	316.324	86.630	1.529	0.209	0.018	0.023	0.013	381.423	27.606	7.111	28.872	203.185	38.709	6.924	5.902	40.551	140.598
Violanthin (μg/g)	1916.484	1119.655	2132.157	1150.442	955.490	1431.087	1591.294	2909.303	2199.678	3002.974	2600.137	1705.626	1859.184	2558.080	1468.573	1112.964	1210.781	2339.195	3822.034	4583.217	1422.127
Vitexin (μg/g)	645.603	291.608	300.206	171.639	178.181	311.331	141.483	1539.759	202.706	1418.220	837.896	408.393	224.438	1446.907	278.403	455.643	175.344	313.920	744.358	1022.246	526.730

Table S8 Regression equations, linear ranges, correlation coefficients and LLOQs of analytes.

No.	Component	Calibration Curve	*R*	Range (ng/mL)	LLOQ (ng/mL)
1	Vitexin	Y=2.038584X+0.023845	0.996	0.0231~1480.00	0.0462
2	Isovitexin	Y=2.661183X+0.010128	0.998	0.0315~2018.80	0.0630
3	Ferulic acid	Y=0.82016X+0.006945	0.997	0.0523~3346.56	0.1046
4	Isoferulic acid	Y=0.082112X-0.00023	0.991	0.1209~7737.60	0.2418
5	Cis-p-coumaric acid	Y=3.428359X+0.173394	0.994	0.0311~2000.00	0.0622
6	Kaempferol-3-β-*O*-glucuronide	Y=1.236311X+0.002697	0.991	0.0311~1991.30	0.0622
7	*N*-trans-feruloyltyramine	Y=1.27156X+1.042404	0.992	0.0011~60.20	0.0022
8	*N*-p-trans-coumaroyltyramine	Y=2.22626X+2.05953	0.997	0.0092~586.00	0.0184

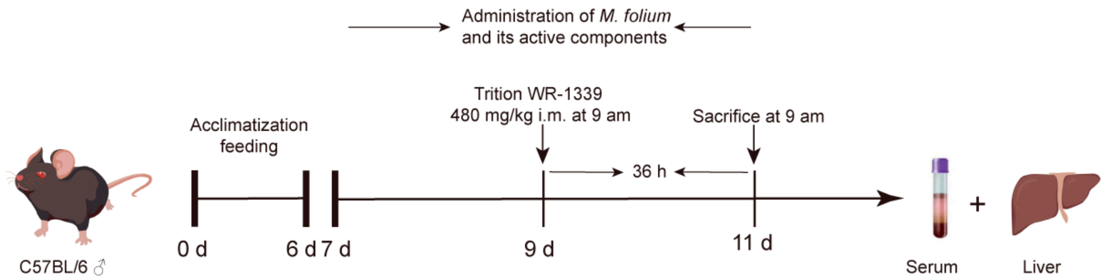

Figure S1 Process of administration of *M. folium* and its active components and hyperlipemia modeling.

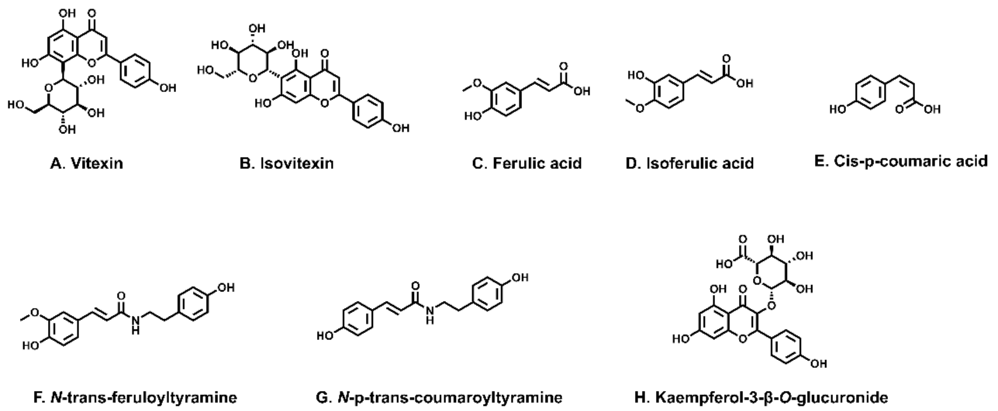

Figure S2 The structural formulas of eight compounds.
